# Conformational signature of Ishikawa´s reagent using NMR information from diastereotopic fluorines

**DOI:** 10.3762/bjoc.15.44

**Published:** 2019-02-20

**Authors:** Laize A F Andrade, Lucas A Zeoly, Rodrigo A Cormanich, Matheus P Freitas

**Affiliations:** 1 Department of Chemistry, Federal University of Lavras, 37200-000, Lavras, MG, Brazil; 2 Institute of Chemistry, University of Campinas, 13083-970, Campinas, SP, Brazil

**Keywords:** anomeric effect, gauche effect, NMR spectroscopy, organofluorine compounds

## Abstract

The active species of the Ishikawa´s reagent [*N*,*N*-diethyl-(1,1,2,3,3,3-hexafluoropropyl)amine] is a fluorinating hexafluoropropylamine used to convert alcohols into alkyl fluorides. On the other hand, it is also an example of model compound useful to probe conformational preferences using spectroscopic information from diastereotopic fluorines. Moreover, the possibility of experiencing both the generalized anomeric and *gauche* effects makes the Ishikawa´s reagent an ideal choice to study the governing stereoelectronic interactions of the conformational equilibrium of organofluorine compounds. The conformational equilibrium of the Ishikawa´s reagent was analyzed using NMR ^3^*J*_H,F_ coupling constant data in different solvents, since the orientation of the diastereotopic fluorines relative to H-2 and F-2 changes with the medium. In nonpolar cyclohexane solvent, the preferred conformation experiences a weaker steric and electrostatic repulsion. The conformational behavior changes in the more polar pyridine solution, where the double fluorine *gauche* effect takes place, since F-2 is preferably *gauche* to both diastereotopic fluorines. An analysis of the rotation around the N–C(F_2_) bond indicates the manifestation of anomeric interactions (*n*_N_ → σ*_C–F_), which can be demonstrated by means of ^19^F chemical shifts. The results were rationalized with the aid of theoretical calculations and natural bond orbital (NBO) analysis, allowing for the evaluation of competing steric, electrostatic and hyperconjugative interactions.

## Introduction

**Figure 1:**
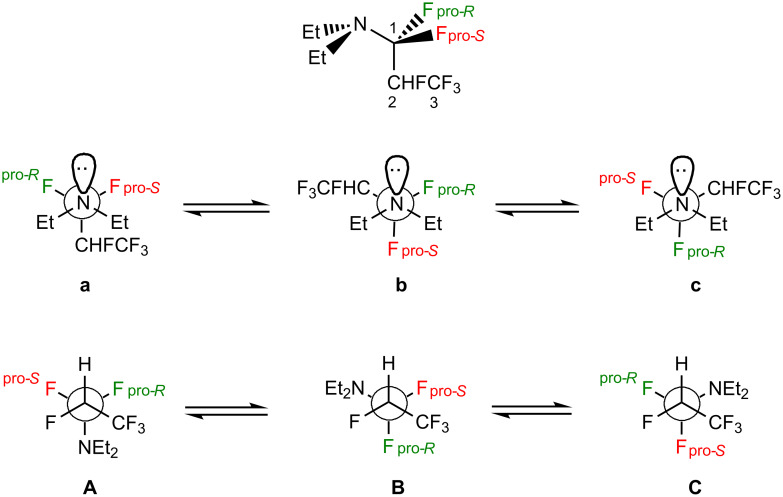
Structure of Ishikawa´s reagent (**1**) and the respective Newman projections indicating the two key rotatable bonds.

The active species of Ishikawa´s reagent [*N*,*N*-diethyl-(1,1,2,3,3,3-hexafluoropropyl)amine, **1**] [1] (Figure 1) has diastereotopic substituents (fluorines), which can be useful to provide conformational insights by using NMR spin–spin coupling constants (SSCCs), such as in methyl 2-fluoroesters [2], 3-fluoro-1,2-propanediol [3], 1-halo-2-propanols [4], enflurane [5], and 1-chloro-1,1-difluoro-2-pentanol [6]. This is possible due to an analogy with the Karplus curve that correlates the magnitude of vicinal ^3^*J*_H,H_ SSCCs with the dihedral angle between coupled nuclei [7]. According to this relationship, the SSCC between antiperiplanar nuclei is larger than that observed between *gauche* nuclei. One-bond SSCCs (^1^*J*) can also provide relevant information on the conformations of a molecule. For example, the Perlin effect manifests in six-membered rings when ^1^*J*_C–Hax_ < ^1^*J*_C–Heq_ [8–9]. A similar effect on ^1^*J*_C–F_ has been observed on fluorinated six-membered rings and then called the (reverse) fluorine Perlin-like effect [10]. Despite some controversies on the origin of such effects [11–14], the anomeric-like interaction *n*_X_ → σ*_C–H/F_ (X = electron donor atom, usually oxygen) seems to contribute to the magnitude of ^1^*J*_C–H/F_, since the resonance structure originated from this interaction exhibits a longer and weaker C–H_ax_/F_ax_ bond relative to C–H_eq_/F_eq_, thus reducing ^1^*J*_C–Hax/Fax_ relative to ^1^*J*_C–Heq/Feq_. In turn, the incoming fluoride becomes magnetically more shielded than the fluorine not involved in such an interaction.

Because the positioning of neighboring groups relative to the diastereotopic fluorines (NEt_2_ and CHFCF_3_ groups), the conformational preferences of Ishikawa´s reagent molecule may be influenced by the generalized anomeric effect, as well as by the fluorine *gauche* effect. The former corresponds to a stabilizing effect originated from the electron delocalization from the nitrogen lone pair to an antiperiplanar C–F antibonding orbital (*n*_N_ → σ*_C–F_), similarly to that observed for some pnictogen compounds and similar systems [15–18]. In turn, the fluorine *gauche* effect may result from the *gauche* orientation between F-1 and F-2, which is sterically and electrostatically disfavored, but it is stabilized by σ_C–H/C–C_ → σ*_C–F_ hyperconjugative interactions [19–23]. Recently, electrostatic polarization, on the basis of the so-called interacting quantum atoms (IQA) method, has been claimed as the origin of the *gauche* effect [24]. These effects have strongly influenced mechanisms of hydrogen exchange and the spectroscopic behavior of a variety of systems [25]; the respective fluorine scenario would then be worth to evaluate.

It is worth mentioning, however, that the relative and interchangeable orientation of the atoms in a molecule (conformations) is dependent on the medium; while only intramolecular interactions drive the conformational stability of a molecule in the vacuum, the solvent polarity plays a significant role in the condensed phase. Thus, it is appropriate to study the conformations of Ishikawa´s reagent in different media, of low and high polarity. This is a challenging task, since the conformational analysis of flexible acyclic organic compounds using NMR SSCCs is more complex than the study of six-membered cyclic compounds, which usually exhibit only two conformations as the result of chair interconversion [26].

## Results and Discussion

The preferred conformation along the H–C2–C1–F fragment in **1** was first analyzed using ^3^*J*_H,F(pro-_*_S_*_/_*_R_*_)_ SSCC data, since such an NMR parameter is sensitive to this dihedral angle according to a Karplus-like shape [27], while its sign is subjected to other effects [28]. Also, the observed ^3^*J*_H,F(pro-_*_S_*_/_*_R_*_)_ values (Table 1) are expected to be dependent on the medium, because the conformers of **1** are anticipated to have different molecular dipole moments and, consequently, the conformer populations are expected to change with the solvent polarity [29]. Indeed, the ^1^H NMR outcomes in C_6_D_12_ (dielectric constant ε = 2.2), CDCl_3_ (ε = 4.8) and C_5_D_5_N (ε = 12.4) solutions are informative on the rotation of H–C2–C1–F and conformational equilibrium in **1**. Some more polar solvents, e.g., MeCN and DMSO, were found to convert Ishikawa´s reagent into an amide; therefore, they were not further studied.

**Table 1:** Experimental coupling constants (Hz) for **1**.

Solvent	^2^*J*_H,F_	^3^*J*_H,Fpro-_*_R_*	^3^*J*_H,Fpro-_*_S_*	^3^*J*_H,F-3_

C_6_D_12_	44.46	11.69	3.53	5.89
CDCl_3_	44.06	11.72	4.04	5.92
C_5_D_5_N	42.34	11.99	6.00	6.00

**Figure 2:**
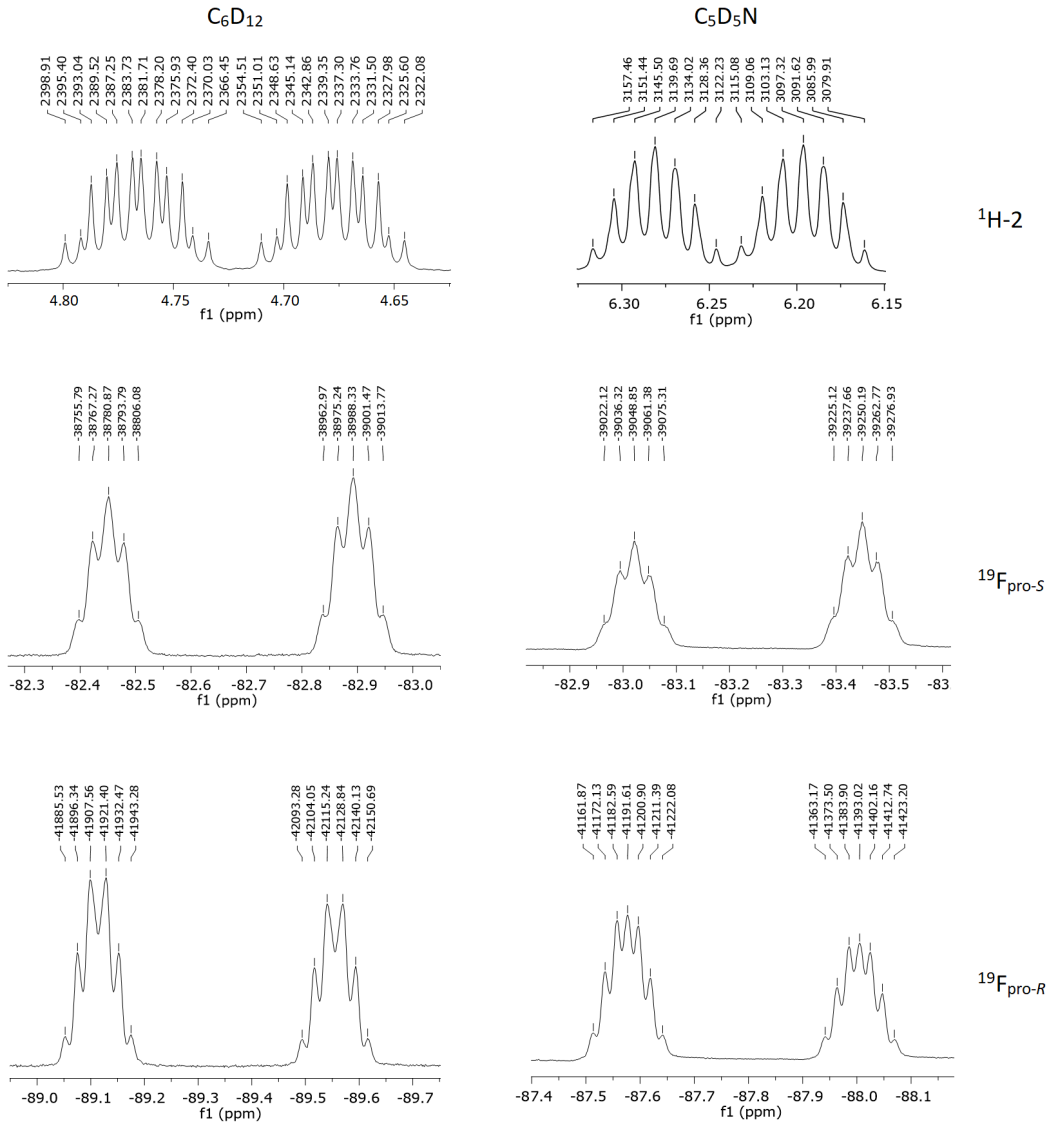
Expansion of the ^1^H and ^19^F NMR spectra in the region of C*H*F and C*F*_2_, in C_6_D_12_ and C_5_D_5_N solvents. The ^19^F chemical shifts were assigned taking into consideration the signal split patterns, coupling constants and possible geometries for the stable conformers.

In nonpolar solution (C_6_D_12_), a *dddq* split pattern appears for H-2 owing to a large ^2^*J*_HF_ of 44.46 Hz, two doublets (11.69 and 3.53 Hz) due to couplings with the diastereotopic fluorines, and a quartet of 5.89 Hz due to the coupling with CF_3_ fluorines (Figure 2). Considering a Karplus-like curve for ^3^*J*_H,F_ SSCCs [27], the magnitude of the ^3^*J*_H,F(pro-_*_S_*_/_*_R_*_)_ SSCCs gives insight into the orientation of the H–C2–C1–F dihedral angles, because the larger value (11.69 Hz) indicates a dominant *anti* orientation for this moiety, while the smaller SSCC (3.53 Hz) would be due to a *gauche* orientation. Accordingly, a dominant contributing conformation regarding the H–C2–C1–F dihedral angle would be expected to be either **1B** or **1C**.

Moreover, there is a subtle solvent dependence of ^3^*J*_H,F(pro-_*_S_*_/_*_R_*_)_, indicating that the conformational equilibrium of **1** changes on going from cyclohexane (nonpolar) to pyridine (polar) solution. According to the calculated molecular dipole moments for the possible conformers of **1** (Table 2), a significant interplay of conformers **1B** and **1C** is not expected when the solvent varies, because of their similar molecular dipole moments. In turn, the populations of conformer **1A** are not prone to increase by increasing the solvent polarity, because of their smaller molecular dipole moments compared to **1B** and **1C**. So, the observed changes in ^3^*J*_H,F(pro-_*_S_*_/_*_R_*_)_ with the solvent is due to a shift from **1A** towards **1B** or **1C**. According to the Karplus curve, conformers **1A** are not anticipated to have significantly different ^3^*J*_H,F(1)_ SSCCs, since both diastereotopic fluorines are *gauche* to H-2. However, SSCC calculations (Supporting Information File 1) show that conformers **1Ab** and **1Ac** exhibit distinct (a small and a large) ^3^*J*_H,F(pro-_*_S_*_)_ and ^3^*J*_H,F(pro-_*_R_*_)_ values, probably as a result of the generalized anomeric effect *n*_N_ → σ*_C-F_ that affects the electron density along the C1–F bond; a smaller ^3^*J*_H,F(1)_ is expected as a C1–F bond is longer and weaker. Because conformer **1Ac** is of very high energy and, therefore, non-populated (Table 2), the larger ^3^*J*_H,F(1)_ SSCC in Table 1 is likely to correspond to ^3^*J*_H,F(pro-_*_R_*_)_, while the smaller one corresponds to ^3^*J*_H,F(pro-_*_S_*_)_. Since the large ^3^*J*_H,F(pro-_*_R_*_)_ SSCC is practically insensitive to solvent changes, while ^3^*J*_H,F(pro-_*_S_*_)_ increases on going from C_6_D_12_ to C_5_D_5_N solution (from 3.53 Hz in cyclohexane solution to 6.00 Hz in pyridine solution), it follows that the above **1A** conformation shifts toward **1C** (F_pro-_*_R_* *anti* in **1B** and *gauche* in **1C** relative to H-2, while F_pro-_*_S_* is *gauche* in **1B** and *anti* in **1C**). The ^3^*J*_F,F_ SSCC does not follow a Karplus-like shape, due to changes in the Fermi contact term with the rotation around the F–C–C–F dihedral angle that even changes the sign [30]; so, the breakdown of the Karplus-like curve and a possible influence of the anomeric effect make this SSCC of little diagnostic value for probing the conformations of **1**. An intermediate conformational behavior is calculated in chloroform solution, because this solvent has a larger dielectric constant than cyclohexane and a smaller value than pyridine, but the experimental ^3^*J*_H,F(1)_ obtained in CDCl_3_ suggests that the conformers population in this solvent is similar to that observed in cyclohexane solution. Thus, further discussion will consider only cyclohexane and pyridine solvents.

**Table 2:** Relative standard Gibbs free energies (in kcal mol^−1^ and Gibbs population in parenthesis) and molecular dipole moments (in Db) for the conformers of **1**, calculated at the ωB97X-D/6-311++g(d,p) level.

Conf.	Cyclohexane		Chloroform		Pyridine	
	
	*G*^0^_rel_ (%)	μ	*G*^0^_rel_ (%)	μ	*G*^0^_rel_ (%)	μ

**1Aa**	1.5 (3)	1.6	1.5 (3)	1.7	1.6 (4)	1.8
**1Ab**	0.0 (40)	2.1	0.4 (20)	2.2	0.9 (15)	2.3
**1Ac**	3.9 (0)	2.5	3.8 (0)	2.6	4.6 (0)	2.7
**1Ba**	1.7 (2)	4.2	1.6 (2)	4.6	2.0 (2)	4.8
**1Bb**	0.5 (17)	4.3	0.3 (21)	4.6	1.0 (12)	4.8
**1Bc**	0.7 (13)	4.5	0.5 (17)	4.9	2.4 (1)	5.2
**1Ca**	4.2 (0)	4.1	3.4 (0)	4.5	4.0 (0)	4.7
**1Cb**	4.0 (0)	4.0	3.9 (0)	4.3	4.2 (0)	4.5
**1Cc**	0.3 (24)	4.2	0.0 (37)	4.6	0.0 (66)	4.9

The generalized anomeric effect (due to the *n*_N_ → σ*_C–F_ hyperconjugation) can be explored for assertion of the C–N–C–F dihedral angle, which contributes to enhance the fluoride character of the fluorine involved in such interaction. Because of the negative charge on the fluorine in the resonance structure derived from the generalized anomeric effect, a shielding effect is expected for this fluorine. The ^19^F NMR assignment of the diastereotopic fluorines was possible considering the ^3^*J*_H,F(1)_ SSCC earlier reported, and comparing the ^19^F and ^19^F{^1^H} NMR experiments: the more shielded diastereotopic fluorine corresponds to F_pro-_*_S_*, thus yielding **1b** as the dominant conformation. Such a shielding effect decreases on going from cyclohexane (−89.4 ppm) to pyridine solution (−87.8 ppm), as the result of a conformational change towards **1a** or **1c**. Since a slight shielding effect is observed on F_pro-_*_R_* on going from cyclohexane to pyridine solution (from −82.7 ppm to −83.2 ppm), an increase in the **1c** conformation is then expected. Because of the rapid relaxation and the subsequent lack of ^13^C-1 signal, the fluorine Perlin effect could not be probed (although the calculated values can be checked in Supporting Information File 1). However, from the NMR results, in general, both **1Ab** and **1Cc** were found to be dominant conformations of the conformational equilibrium of the Ishikawa´s reagent. In addition, this equilibrium shifts from **1Ab** to **1Cc** when increasing the solvent polarity. These findings are in complete agreement with the conformational energy data provided in Table 2, which were obtained from high level DFT calculations (**1Cb** is not a minimum-energy conformer). The DFT results are also consistent with ab initio MP2 electronic energies (Supporting Information File 1).

Opposite to the expectation of a double fluorine *gauche* effect (σ_C–H/C–C_ → σ*_C–F_) [19–23] as ruling mechanism of the conformational stability of **1**, the **1Ab** conformer appears as the main conformer in a nonpolar medium. In part, the hyperconjugative interaction above (which is possible in **1B** and **1C** conformers) is somewhat counterbalanced by an σ_C–H_ → σ*_C–N_ interaction in **1A**, since σ*_C–N_ is also a good electron acceptor orbital (see NBO energies in Table 3). In addition, this conformation avoids exceedingly strong dipolar repulsions due to two C–F/C–F Coulombic contacts, such as in **1C**. In turn, the double *gauche* effect (in which F-2 is *gauche* to both diastereotopic fluorines) takes place in a more polar solution (due to **1C**), as the dipolar repulsion between the vicinal fluorines is attenuated by the polar solvent, while the highly stabilizing hyperconjugative interactions are evidenced.

**Table 3:** Natural bond orbital (NBO) energies (in kcal mol^−1^) for **1** in implicit cyclohexane and pyridine (second entries, in parenthesis) solutions.

Interaction	**1Aa**	**1Ab**	**1Ac**	**1Ba**	**1Bb**	**1Bc**	**1Ca**	**1Cb**	**1Cc**

*n*_N_ → σ*_C–F(pro-_*_R_*_)_	4.0 (4.0)	3.4 (3.6)	– (–)	4.8 (4.8)	5.1 (5.6)	34.4 (36.6)	17.4 (18.1)	0.5 –	34.2 (35.1)
*n*_N_ → σ*_C–F(pro-_*_S_*_)_	15.7 (16.0)	35.2 (36.6)	– (–)	13.1 (13.6)	36.7 (38.0)	9.3 (10.3)	2.0 (2.1)	23.0 (24.6)	2.3 (2.7)
*n*_N_ → σ*_C1–C2_	17.7 (17.8)	3.7 (3.6)	– (–)	16.0 (16.2)	2.8 (2.7)	1.4 (1.3)	14.7 (15.0)	8.3 (8.5)	4.0 (3.8)
σ_C–H_ → σ*_C–F(pro-_*_R_*_)_	1.0 (1.0)	1.8 (1.8)	0.6 (0.5)	4.6 (4.7)	4.5 (4.6)	4.2 (4.3)	0.9 (0.9)	1.4 (1.4)	0.9 (0.9)
σ_C–H_ → σ*_C–F(pro-_*_S_*_)_	0.9 (0.9)	0.5 (0.5)	1.4 (1.5)	0.8 (0.7)	1.2 (1.1)	– (–)	4.6 (4.7)	4.8 (4.8)	4.2 (4.1)
σ_C–H_ → σ*_C–N_	3.6 (3.7)	4.0 (4.1)	4.6 (4.5)	– (–)	– (–)	0.5 (0.5)	– (–)	– (–)	– (–)
σ_C–F2_ → σ*_C–F(pro-_*_R_*_)_	1.3 (1.4)	1.2 (1.2)	0.7 (0.7)	– (–)	– (–)	– (–)	– (–)	– (–)	– (–)
σ_C–F2_ → σ*_C–F(pro-_*_S_*_)_	– (–)	– (–)	– (–)	1.4 (1.5)	1.3 (1.4)	1.5 (1.5)	– (–)	– (–)	– (–)
σ_C–F2_ → σ*_C–N_	– (–)	– (–)	0.6 (0.6)	– (–)	– (–)	– (–)	1.2 (1.3)	1.4 (1.4)	1.5 (1.5)
σ_C2–C3_ → σ*_C–F(pro-_*_R_*_)_	– (–)	– (–)	– (–)	– (–)	0.7 (0.7)	– (–)	1.8 (1.8)	1.6 (1.6)	2.2 (2.2)
σ_C–C3_ → σ*_C–F(pro-_*_S_*_)_	1.6 (1.6)	2.0 (2.0)	1.2 (1.3)	– (–)	– (–)	0.7 (0.7)	– (–)	– (–)	– (–)
σ_C–C3_ → σ*_C–N_	– (–)	– (–)	– (–)	1.5 (1.5)	1.8 (1.8)	1.7 (1.7)	– (–)	– (–)	– (–)

## Conclusion

*N*,*N*-Diethyl-(1,1,2,3,3,3-hexafluoropropyl)amine (**1**) experiences the generalized anomeric effect in both nonpolar and polar solvents and, therefore, the *n*_N_ → σ*_C–F_ hyperconjugative interaction plays a determinant role for the rotation around the N–C(F_2_) bond in all tested media. However, the conformers capable of maximally performing the fluorine *gauche* effect, which is widely known to be due to an antiperiplanar σ_C–H/C–C_ → σ*_C–F_ orbital interaction in similar systems, are not dominant in cyclohexane solution. Such an effect is actually manifested totally (as a double *gauche* effect, due to the *gauche* orientation of F-2 relative to both diastereotopic fluorines) in a polar solvent, where the dipolar repulsion is attenuated, and the *gauche* effect interactions then override the repulsive forces. Because of the significant difference in the molecular dipole moments for the conformers of **1**, their populations were sensitive to solvent changes. Since the vicinal ^3^*J*_H2,F1_ spin-spin coupling constants were found to be conformation-dependent, as well as the ^19^F chemical shifts, these NMR parameters provided detailed account on the H–C2–C1–F dihedral angle as the solvent varied.

## Experimental and Computational Details

*N*,*N*-Diethyl-(1,1,2,3,3,3-hexafluoropropyl)amine (**1**) was commercially available (90% purity) and used without further purification. The NMR spectra were acquired at 400.2 or 499.9 MHz for ^1^H, 470.3 MHz for ^19^F, and 125.7 MHz for ^13^C, from ca. 10 mg mL^−1^ solutions in C_6_D_12_, CDCl_3_ and C_5_D_5_N solvents. The geometries for the conformers of **1** were fully optimized (including frequency calculations) at the ωB97X-D/6-311++G(d,p) level [31–32], which includes some empirical dispersion effects. The calculations were carried out considering both the gas phase and implicit solvation, according to the polarizable continuum model [33]. The nine possible conformers were selected after a previous screening from 81 structures, which differed by the orientation of the *N*-ethyl groups. Subsequent natural bond orbital (NBO) [34] analyses at the ωB97X-D/6-311++G(d,p) level [31–32] were performed to obtain the second-order perturbation energies of donor–acceptor interactions. This same level of theory was employed for the chemical shift and SSCC calculations.

## Supporting Information

File 1 Standard coordinates for the geometries of conformers of **1**, NMR spectra and tables containing calculated spectroscopic data.
