# Supplementary material for: Conformational signature of Ishikawa´s reagent using NMR information from diastereotopic fluorines
Source: Beilstein J Org Chem. 2019 Feb 20;15:506–12. doi: 10.3762/bjoc.15.44 (PMC6404478; doi:10.3762/bjoc.15.44)
Supplement: File 1 — Standard coordinates for the geometries of conformers of 1, NMR spectra and tables containing calculated spectroscopic data. [file Beilstein_J_Org_Chem-15-506-s001.pdf]

BEILSTEIN JOURNAL OF ORGANIC CHEMISTRY

Supporting Information

for

Conformational signature of Ishikawa´s reagent using NMR information from diastereotopic fluorines

Laize A. F. Andrade, Lucas A. Zeoly, Rodrigo A. Cormanich and Matheus P. Freitas

Beilstein J. Org. Chem. **2019,** *15,* 506–512. doi:10.3762/bjoc.15.44

Standard coordinates for the geometries of conformers of 1, NMR spectra and tables containing calculated spectroscopic data

License and Terms: This is a supporting information file under the terms of the Creative Commons Attribution License (http://creativecommons.org/licenses/by/4.0). Please note that the reuse, redistribution and reproduction in particular requires that the authors and source are credited.
The license is subject to the *Beilstein Journal of Organic Chemistry* terms and conditions: (https://www.beilstein-journals.org/bjoc)

Contents

Pages S2–S15. Standard coordinates for the conformers of **1**, optimized in implicit cyclohexane, chloroform and pyridine solvents at the ωB97X-D/6-311++g(d,p) level.

Page S16. Table S1. Calculated [ωB97X-D/6-311++g(d,p)] spin–spin coupling constants and F–C–C–F dihedral angles, in implicit cyclohexane solvent.

Page S17. Table S2. Calculated [ωB97X-D/6-311++g(d,p)] spin–spin coupling constants and F–C–C–F dihedral angles, in implicit pyridine solvent.

Page S18. Table S3. Calculated $^{3}J_{H2,F1}$ coupling constants (in Hz) and H–C–C–F(1) dihedral angles (in degrees) for the optimized geometries of **1**.

Page S19. Table S4. Relative nuclear-electronic energies (in kcal mol^{-1} and population % in parenthesis) and molecular dipole moments (in D) for the conformers of **1**, calculated at the MP2/6-311++g(d,p) level.

Page S20–S31. Figures S1 to S12. ^{1}H, ^{19}F and ^{13}C NMR spectra for **1**, in C_6D_{12}, $CDCl_3$ and C_5D_5N solutions.

1Aa_C_6H_{12}_ωB97X-D/6-311++G(d,p) *E*= −927.1983983

Standard orientation:

Center Number	Atomic Number	Atomic Type	Coordinates (Angstroms) X	Y	Z
1	6	0	-2.678614	0.940613	0.000000
2	1	0	-3.035286	0.437909	-0.874628
3	1	0	-3.035287	0.434522	0.872672
4	6	0	-0.625272	1.666550	1.257405
5	1	0	-0.982252	1.162369	2.131056
6	1	0	0.444728	1.666211	1.257593
7	6	0	-0.625298	-0.511338	0.000000
8	9	0	0.724702	-0.511354	-0.000321
9	9	0	-1.075568	-1.147821	-1.102110
10	7	0	-1.138614	0.940594	0.000000
11	6	0	-1.138341	-1.237189	1.257588
12	1	0	-0.800791	-2.252486	1.246050
13	6	0	-2.677974	-1.208267	1.274786
14	9	0	-3.155545	-2.351529	0.738709
15	9	0	-3.116365	-0.155196	0.552737
16	9	0	-3.111688	-1.092723	2.547987
17	9	0	-0.664291	-0.617860	2.359498
18	6	0	-1.138144	3.118639	1.257135
19	1	0	-0.526904	3.712907	1.903765
20	1	0	-1.093817	3.512370	0.263197
21	1	0	-2.150059	3.139559	1.604254
22	6	0	-3.191929	2.392543	0.002816
23	1	0	-3.345348	2.714647	1.011583
24	1	0	-2.470399	3.026847	-0.468303
25	1	0	-4.116693	2.444942	-0.532876

1Ab_C_6H_{12}_ωB97X-D/6-311++G(d,p) *E* = −927.2009632

Standard orientation:

Center Number	Atomic Number	Atomic Type	Coordinates (Angstroms) X	Y	Z
1	6	0	-3.479231	0.094755	1.224585
2	1	0	-3.685767	-0.666872	0.475765
3	1	0	-4.161005	-0.095768	2.064559
4	6	0	-1.731559	0.814619	2.826838
5	1	0	-2.653831	1.264804	3.203321
6	1	0	-1.341882	0.201117	3.641905
7	6	0	-1.551998	-1.319250	1.702217
8	9	0	-2.171328	-2.166364	2.625095
9	9	0	-0.254985	-1.260706	2.124186
10	7	0	-2.087412	-0.038359	1.675851
11	6	0	-1.512034	-2.135172	0.388218
12	1	0	-0.823070	-2.963043	0.569033
13	6	0	-1.001501	-1.373366	-0.841916
14	9	0	0.145841	-0.746374	-0.580681
15	9	0	-1.879071	-0.475915	-1.293900
16	9	0	-0.775954	-2.243769	-1.836652
17	9	0	-2.739654	-2.658827	0.071526
18	6	0	-0.727793	1.894810	2.451302
19	1	0	-0.514930	2.532609	3.313744
20	1	0	0.208175	1.446750	2.112669
21	1	0	-1.116452	2.520122	1.644373
22	6	0	-3.746878	1.467877	0.626207
23	1	0	-3.601070	2.266201	1.357978
24	1	0	-3.083505	1.651443	-0.220769
25	1	0	-4.781008	1.522310	0.278336

1Ac_C_6H_{12}_ωB97X-D/6-311++G(d,p) $E = -927.1943787$

Standard orientation:

Center Number	Atomic Number	Atomic Type	Coordinates (Angstroms) X	Y	Z
1	6	0	-2.606598	-0.470148	-0.032288
2	1	0	-3.324306	0.085095	-0.641089
3	1	0	-2.655082	-1.504220	-0.372813
4	6	0	-0.974729	1.424767	0.008464
5	1	0	-0.893767	1.555692	1.094697
6	1	0	-0.015208	1.694459	-0.426138
7	6	0	-0.276469	-0.920194	-0.418651
8	9	0	-0.573945	-1.820960	-1.422474
9	9	0	-0.193964	-1.751554	0.704057
10	7	0	-1.264907	0.035757	-0.342199
11	6	0	1.180860	-0.495927	-0.686365
12	1	0	1.706904	-1.434482	-0.867625
13	6	0	1.957506	0.221358	0.458263
14	9	0	3.123830	-0.408251	0.658735
15	9	0	1.305174	0.230373	1.621464
16	9	0	2.238807	1.486030	0.139419
17	9	0	1.257107	0.263645	-1.820959
18	6	0	-2.014405	2.385469	-0.553928
19	1	0	-1.686728	3.412076	-0.376686
20	1	0	-2.131275	2.240053	-1.630281
21	1	0	-2.990519	2.266727	-0.078475
22	6	0	-2.983706	-0.386857	1.444469
23	1	0	-2.276918	-0.947939	2.058102
24	1	0	-3.005602	0.646493	1.800806
25	1	0	-3.980992	-0.808614	1.592929

1Ba_C_6H_{12}_ωB97X-D/6-311++G(d,p) $E = -927.1975755$

Standard orientation:

Center Number	Atomic Number	Atomic Type	Coordinates (Angstroms) X	Y	Z
1	6	0	-2.289275	1.102516	0.808931
2	1	0	-2.080364	0.611894	-0.139235
3	1	0	-3.247826	0.709787	1.168647
4	6	0	-1.326892	1.290554	3.103948
5	1	0	-0.500473	0.885183	3.689225
6	1	0	-1.156100	2.369708	3.066330
7	6	0	-0.534332	-0.450748	1.589693
8	9	0	0.712898	-0.357321	2.154214
9	9	0	-0.347913	-0.714904	0.272202
10	7	0	-1.203797	0.772819	1.737689
11	6	0	-1.194128	-1.684802	2.257290
12	1	0	-1.167098	-1.540410	3.340300
13	6	0	-0.573707	-3.057089	1.965390
14	9	0	0.713348	-3.089094	2.317218
15	9	0	-0.670239	-3.405471	0.684146
16	9	0	-1.214696	-3.991494	2.686005
17	9	0	-2.506710	-1.744947	1.850215
18	6	0	-2.656905	1.013227	3.805369
19	1	0	-2.608956	1.390026	4.829759
20	1	0	-3.488866	1.516434	3.307500
21	1	0	-2.891395	-0.053058	3.846112
22	6	0	-2.390431	2.605025	0.583661
23	1	0	-2.679154	3.138330	1.493143
24	1	0	-1.435800	3.006430	0.236286
25	1	0	-3.150998	2.810819	-0.172995

1Bb_C_6H_{12}_ωB97X-D/6-311++G(d,p) $E = -927.1988454$

Standard orientation:

Center Number	Atomic Number	Atomic Type	Coordinates (Angstroms) X	Y	Z
1	6	0	-3.435396	0.653620	1.502306
2	1	0	-3.735065	0.088614	0.620127
3	1	0	-4.183702	0.451103	2.281252
4	6	0	-1.642429	0.775815	3.213283
5	1	0	-2.491993	1.277385	3.685535
6	1	0	-1.348179	-0.024632	3.895247
7	6	0	-1.789423	-1.135074	1.745163
8	9	0	-2.498388	-2.014651	2.579892
9	9	0	-0.487966	-1.338456	2.087180
10	7	0	-2.114692	0.196779	1.941806
11	6	0	-2.049965	-1.664721	0.316554
12	1	0	-3.112312	-1.891258	0.209934
13	6	0	-1.283702	-2.948656	-0.042667
14	9	0	-1.473629	-3.906686	0.864557
15	9	0	0.023835	-2.742556	-0.177228
16	9	0	-1.740834	-3.405844	-1.219720
17	9	0	-1.697676	-0.721680	-0.613185
18	6	0	-0.488045	1.748495	3.018521
19	1	0	-0.183058	2.170050	3.980321
20	1	0	0.369814	1.238399	2.576902
21	1	0	-0.774643	2.568494	2.356660
22	6	0	-3.435866	2.131843	1.140634
23	1	0	-3.203849	2.761129	2.002938
24	1	0	-2.701269	2.332355	0.358009
25	1	0	-4.424748	2.420419	0.777077

1Bc_C_6H_{12}_ωB97X-D/6-311++G(d,p) $E = -927.198922$

Standard orientation:

Center Number	Atomic Number	Atomic Type	Coordinates (Angstroms) X	Y	Z
1	6	0	-3.666243	-0.190422	1.421050
2	1	0	-3.940250	-1.238865	1.306997
3	1	0	-4.250499	0.191301	2.264692
4	6	0	-1.843276	1.071731	2.522350
5	1	0	-0.769318	1.239451	2.415423
6	1	0	-2.324447	1.925861	2.040264
7	6	0	-1.582799	-1.312833	1.938149
8	9	0	-1.834764	-2.142178	0.878309
9	9	0	-2.054646	-2.026145	3.034579
10	7	0	-2.231348	-0.101598	1.734647
11	6	0	-0.048577	-1.194647	2.088681
12	1	0	0.319391	-0.431629	1.399480
13	6	0	0.729492	-2.484487	1.774737
14	9	0	0.734229	-2.724191	0.460190
15	9	0	0.251450	-3.558505	2.398479
16	9	0	2.002565	-2.323069	2.158393
17	9	0	0.266423	-0.828422	3.372423
18	6	0	-2.219265	1.031841	4.002955
19	1	0	-1.950604	1.981396	4.472619
20	1	0	-3.292890	0.883570	4.139258
21	1	0	-1.693950	0.231053	4.523650
22	6	0	-4.006681	0.570220	0.146925
23	1	0	-3.729403	1.624776	0.224695
24	1	0	-3.478012	0.138874	-0.705491
25	1	0	-5.081382	0.520679	-0.046338

1Ca_C_6H_{12}_ωB97X-D/6-311++G(d,p) $E = -927.1949454$

Standard orientation:

Center Number	Atomic Number	Atomic Type	Coordinates (Angstroms) X	Y	Z
1	6	0	-1.665312	-1.372149	-0.844694
2	1	0	-2.704198	-1.244634	-1.167493
3	1	0	-1.056658	-1.349149	-1.747381
4	6	0	-2.424695	0.286849	0.809223
5	1	0	-3.075880	0.914894	0.183562
6	1	0	-3.020594	-0.586483	1.081546
7	6	0	-0.278112	0.623045	-0.378183
8	9	0	0.633689	-0.053882	-1.118184
9	9	0	0.406738	1.088317	0.720132
10	7	0	-1.302057	-0.245921	0.028156
11	6	0	-0.671428	1.911788	-1.144667
12	1	0	-1.331151	2.506040	-0.507097
13	6	0	-1.399857	1.699992	-2.476780
14	9	0	-2.607256	1.150844	-2.272302
15	9	0	-0.731648	0.917394	-3.323195
16	9	0	-1.592123	2.879246	-3.075271
17	9	0	0.454895	2.646880	-1.411654
18	6	0	-2.056677	1.024050	2.090156
19	1	0	-2.974211	1.233985	2.645158
20	1	0	-1.400970	0.414419	2.713888
21	1	0	-1.556492	1.975483	1.903948
22	6	0	-1.485482	-2.709993	-0.140631
23	1	0	-0.434705	-2.870030	0.108858
24	1	0	-2.066116	-2.750767	0.784968
25	1	0	-1.820260	-3.525777	-0.787058

1Cb_C_6H_{12}_ωB97X-D/6-311++G(d,p) $E = -927.1947101$

Center Number	Atomic Number	Atomic Type	Coordinates (Angstroms) X	Y	Z
1	7	0	-1.796216	2.212106	1.733944
2	6	0	-1.203616	3.190292	2.664365
3	1	0	-0.684881	2.689038	3.485550
4	1	0	-2.035331	3.731822	3.116805
5	6	0	-0.280374	4.175768	1.962729
6	1	0	0.101055	4.912991	2.674375
7	1	0	-0.820634	4.702493	1.172197
8	1	0	0.574545	3.664561	1.515246
9	6	0	-3.963644	3.326821	1.263202
10	1	0	-3.690506	3.487423	0.219609
11	1	0	-3.713889	4.230046	1.825312
12	1	0	-5.046024	3.189877	1.324018
13	6	0	-3.259678	2.098722	1.821575
14	1	0	-3.582851	1.217081	1.271048
15	1	0	-3.556402	1.928694	2.864998
16	6	0	-1.093361	1.008633	1.537353
17	9	0	0.173294	1.124631	2.024523
18	9	0	-1.647934	-0.056045	2.212539
19	6	0	-0.875753	0.586719	0.062632
20	1	0	-0.250901	1.357755	-0.394826
21	6	0	-2.096951	0.400008	-0.844532
22	9	0	-1.699259	-0.100761	-2.018166
23	9	0	-3.004684	-0.437622	-0.335726
24	9	0	-2.700125	1.568639	-1.091502
25	9	0	-0.193860	-0.604297	0.050182

1Cc_C_6H_{12}_ωB97X-D/6-311++G(d,p) *E* = −927.2000985

Standard orientation:

Center Number	Atomic Number	Atomic Type	Coordinates (Angstroms) X	Y	Z
1	6	0	-3.639171	0.660171	1.758059
2	1	0	-4.097733	-0.311354	1.564266
3	1	0	-4.113833	1.043903	2.665086
4	6	0	-1.766272	1.045333	3.329580
5	1	0	-2.361314	0.608673	4.143073
6	1	0	-0.730229	0.759976	3.512839
7	6	0	-1.661614	-0.738473	1.673281
8	9	0	-2.193857	-1.139003	0.492779
9	9	0	-1.980199	-1.769605	2.564241
10	7	0	-2.199669	0.492294	2.040848
11	6	0	-0.123039	-0.792371	1.540687
12	1	0	0.347116	-0.711566	2.521762
13	6	0	0.486835	0.297651	0.645503
14	9	0	0.486178	1.479298	1.277433
15	9	0	-0.146010	0.446811	-0.515754
16	9	0	1.761394	-0.011070	0.379634
17	9	0	0.210112	-2.008472	1.007275
18	6	0	-1.863632	2.563456	3.357237
19	1	0	-1.512720	2.936597	4.322122
20	1	0	-1.251693	3.001513	2.567087
21	1	0	-2.893118	2.904115	3.225511
22	6	0	-3.886595	1.603016	0.589623
23	1	0	-3.453584	2.586219	0.787442
24	1	0	-3.434735	1.207792	-0.322001
25	1	0	-4.960156	1.723118	0.420420

1Aa_$CHCl_3$_ωB97X-D/6-311++G(d,p) *E* = −927.1998691

Standard orientation:

Center Number	Atomic Number	Atomic Type	Coordinates (Angstroms) X	Y	Z
1	6	0	-1.389626	-1.291010	0.522379
2	1	0	-0.496899	-1.900119	0.371620
3	1	0	-1.503975	-1.182486	1.604574
4	6	0	-1.528216	1.187057	0.829390
5	1	0	-0.952883	1.135096	1.763576
6	1	0	-1.230691	2.102389	0.318822
7	6	0	-0.197062	0.243027	-0.993480
8	9	0	-0.558713	1.270934	-1.833455
9	9	0	-0.092148	-0.873523	-1.767513
10	7	0	-1.192363	0.052933	-0.034890
11	6	0	1.263075	0.610322	-0.587230
12	1	0	1.877689	0.631987	-1.489656
13	6	0	1.934759	-0.328980	0.417150
14	9	0	1.977285	-1.583427	-0.043499
15	9	0	1.308922	-0.340692	1.598877
16	9	0	3.193544	0.068136	0.635198
17	9	0	1.266022	1.861186	-0.016913
18	6	0	-3.017743	1.248078	1.137801
19	1	0	-3.230371	2.126980	1.751166
20	1	0	-3.597875	1.316084	0.214753
21	1	0	-3.352325	0.367755	1.692130
22	6	0	-2.595608	-2.007679	-0.072500
23	1	0	-3.510648	-1.432612	0.083384
24	1	0	-2.461210	-2.153131	-1.146091
25	1	0	-2.719455	-2.988133	0.396221

1Ab_$CHCl_3$_ωB97X-D/6-311++G(d,p) $E = -927.2024633$

Standard orientation:

Center Number	Atomic Number	Atomic Type	Coordinates (Angstroms) X	Y	Z
1	6	0	1.194346	1.169730	1.120673
2	1	0	0.196022	1.346204	1.515064
3	1	0	1.834770	0.930827	1.980305
4	6	0	2.477797	-0.446463	-0.252863
5	1	0	3.235280	0.089179	0.324646
6	1	0	2.605275	-1.503795	-0.011472
7	6	0	0.234360	-0.977331	0.483756
8	9	0	0.484065	-1.647382	1.688184
9	9	0	0.322294	-1.957646	-0.462485
10	7	0	1.153666	0.022777	0.202214
11	6	0	-1.267926	-0.610279	0.546899
12	1	0	-1.814237	-1.554332	0.498187
13	6	0	-1.771827	0.271880	-0.602780
14	9	0	-1.406398	-0.222841	-1.786358
15	9	0	-1.336269	1.530106	-0.519901
16	9	0	-3.111886	0.314404	-0.572139
17	9	0	-1.594598	0.015721	1.723846
18	6	0	2.686194	-0.216047	-1.742564
19	1	0	3.686990	-0.540913	-2.040033
20	1	0	1.953036	-0.778417	-2.323993
21	1	0	2.577438	0.842903	-1.987963
22	6	0	1.689213	2.430493	0.427958
23	1	0	2.707926	2.313595	0.050716
24	1	0	1.038993	2.689451	-0.409613
25	1	0	1.691779	3.260157	1.138563

1Ac_$CHCl_3$_ωB97X-D/6-311++G(d,p) $E = -927.1960818$

Standard orientation:

Center Number	Atomic Number	Atomic Type	Coordinates (Angstroms) X	Y	Z
1	6	0	2.595521	-0.494662	-0.089377
2	1	0	3.405257	-0.004416	0.456046
3	1	0	2.663644	-1.554790	0.152078
4	6	0	1.009969	1.415383	0.204913
5	1	0	0.824757	1.603143	-0.858804
6	1	0	0.100195	1.667287	0.744411
7	6	0	0.338453	-0.940785	0.616777
8	9	0	0.782736	-1.921904	1.487792
9	9	0	0.041068	-1.692396	-0.531630
10	7	0	1.320027	0.004603	0.444064
11	6	0	-1.038403	-0.518705	1.164325
12	1	0	-1.542711	-1.454141	1.409325
13	6	0	-1.995193	0.251277	0.204664
14	9	0	-3.152464	-0.416987	0.103026
15	9	0	-1.515177	0.397489	-1.031959
16	9	0	-2.276393	1.467204	0.677217
17	9	0	-0.885384	0.198547	2.320805
18	6	0	2.111760	2.335697	0.712226
19	1	0	1.787787	3.372825	0.602801
20	1	0	2.317858	2.145608	1.768011
21	1	0	3.040596	2.221006	0.149569
22	6	0	2.768524	-0.289960	-1.591616
23	1	0	1.955822	-0.763354	-2.145829
24	1	0	2.796904	0.770002	-1.856530
25	1	0	3.712394	-0.736427	-1.913153

1Ba_$CHCl_3$_ωB97X-D/6-311++G(d,p) $E = -927.1996028$

Standard orientation:

Center Number	Atomic Number	Atomic Type	Coordinates (Angstroms) X	Y	Z
1	6	0	2.225895	-0.654591	-0.890175
2	1	0	1.730604	-1.543547	-1.274633
3	1	0	2.134059	0.122022	-1.658573
4	6	0	2.029170	0.968758	0.996996
5	1	0	1.364777	1.186859	1.834085
6	1	0	3.000280	0.742403	1.444513
7	6	0	0.178776	-0.535922	0.486277
8	9	0	-0.141233	-0.584841	1.821369
9	9	0	-0.091141	-1.766700	-0.021055
10	7	0	1.544405	-0.254596	0.346065
11	6	0	-0.808587	0.484561	-0.135570
12	1	0	-0.698544	1.434082	0.393547
13	6	0	-2.298725	0.121501	-0.116749
14	9	0	-2.732867	-0.078595	1.129553
15	9	0	-2.575609	-0.964976	-0.837541
16	9	0	-3.007488	1.136877	-0.635244
17	9	0	-0.460390	0.666867	-1.454605
18	6	0	2.161655	2.196274	0.095829
19	1	0	2.471448	3.051659	0.700537
20	1	0	2.916783	2.049411	-0.679586
21	1	0	1.222119	2.453896	-0.398657
22	6	0	3.693984	-0.969470	-0.638205
23	1	0	4.256903	-0.084831	-0.329886
24	1	0	3.796327	-1.731996	0.137317
25	1	0	4.148734	-1.344808	-1.557703

1Bb_$CHCl_3$_ωB97X-D/6-311++G(d,p) $E = -927.2010029$

Standard orientation:

Center Number	Atomic Number	Atomic Type	Coordinates (Angstroms) X	Y	Z
1	6	0	-3.440527	0.652718	1.507899
2	1	0	-3.745712	0.081941	0.631627
3	1	0	-4.189464	0.463411	2.288772
4	6	0	-1.644576	0.772970	3.219668
5	1	0	-2.492822	1.274763	3.693577
6	1	0	-1.344550	-0.023394	3.903769
7	6	0	-1.792695	-1.138037	1.748779
8	9	0	-2.491708	-2.031263	2.581832
9	9	0	-0.487425	-1.338696	2.087268
10	7	0	-2.122929	0.188545	1.952891
11	6	0	-2.051461	-1.661508	0.317944
12	1	0	-3.113718	-1.882800	0.204402
13	6	0	-1.285776	-2.943544	-0.047088
14	9	0	-1.473682	-3.906213	0.856020
15	9	0	0.023439	-2.736805	-0.179578
16	9	0	-1.739494	-3.396928	-1.226269
17	9	0	-1.689681	-0.714362	-0.605849
18	6	0	-0.494254	1.748839	3.015174
19	1	0	-0.190204	2.179134	3.973231
20	1	0	0.365867	1.239628	2.576427
21	1	0	-0.786212	2.562339	2.347678
22	6	0	-3.426431	2.128307	1.136056
23	1	0	-3.188347	2.760294	1.994735
24	1	0	-2.688936	2.316860	0.352961
25	1	0	-4.411885	2.424087	0.769170

1Bc_$CHCl_3$_ωB97X-D/6-311++G(d,p) $E = -927.2010494$

Standard orientation:

Center Number	Atomic Number	Atomic Type	Coordinates (Angstroms) X	Y	Z
1	6	0	-2.614053	-0.850000	0.363280
2	1	0	-2.215775	-1.465484	1.169609
3	1	0	-3.314630	-0.148850	0.827281
4	6	0	-1.889256	1.199769	-0.826053
5	1	0	-1.130639	1.515360	-1.545332
6	1	0	-2.792768	1.016661	-1.411912
7	6	0	-0.264090	-0.264421	0.319571
8	9	0	0.011202	-1.602970	0.428505
9	9	0	-0.185815	0.189163	1.637091
10	7	0	-1.511695	-0.098166	-0.258658
11	6	0	0.896640	0.395158	-0.460209
12	1	0	0.719482	0.283516	-1.531622
13	6	0	2.283369	-0.207106	-0.175572
14	9	0	2.401994	-1.412659	-0.738871
15	9	0	2.556643	-0.321355	1.123756
16	9	0	3.218014	0.584261	-0.717681
17	9	0	0.962280	1.731085	-0.153004
18	6	0	-2.144831	2.313692	0.187842
19	1	0	-2.490157	3.209226	-0.334565
20	1	0	-2.914916	2.028891	0.908342
21	1	0	-1.237361	2.565945	0.737163
22	6	0	-3.332297	-1.729187	-0.650822
23	1	0	-3.729551	-1.137525	-1.479640
24	1	0	-2.648358	-2.476317	-1.058921
25	1	0	-4.170317	-2.245203	-0.175306

1Ca_$CHCl_3$_ωB97X-D/6-311++G(d,p) $E = -927.1969183$

Standard orientation:

Center Number	Atomic Number	Atomic Type	Coordinates (Angstroms) X	Y	Z
1	6	0	-1.650172	-1.371434	-0.847185
2	1	0	-2.678239	-1.236233	-1.199539
3	1	0	-1.015382	-1.367901	-1.731535
4	6	0	-2.417950	0.289683	0.805356
5	1	0	-3.058460	0.936160	0.188740
6	1	0	-3.024023	-0.582746	1.054956
7	6	0	-0.278640	0.636649	-0.386862
8	9	0	0.642290	-0.032756	-1.125931
9	9	0	0.407720	1.108904	0.710704
10	7	0	-1.292749	-0.239573	0.022281
11	6	0	-0.679590	1.921473	-1.154815
12	1	0	-1.341377	2.516162	-0.520502
13	6	0	-1.403360	1.699192	-2.487831
14	9	0	-2.606478	1.144088	-2.286838
15	9	0	-0.725833	0.916423	-3.328917
16	9	0	-1.598152	2.874260	-3.094562
17	9	0	0.446054	2.660221	-1.425221
18	6	0	-2.049912	0.996292	2.103457
19	1	0	-2.967116	1.195165	2.662761
20	1	0	-1.397622	0.368834	2.713462
21	1	0	-1.546494	1.950184	1.939970
22	6	0	-1.507636	-2.701703	-0.120393
23	1	0	-0.468950	-2.864526	0.174284
24	1	0	-2.127692	-2.729468	0.779702
25	1	0	-1.820795	-3.522914	-0.770534

1Cb_$CHCl_3$_ωB97X-D/6-311++G(d,p) $E = -927.1965531$

Center Number	Atomic Number	Atomic Type	Coordinates (Angstroms) X	Y	Z
1	7	0	-1.796147	2.212319	1.736062
2	6	0	-1.206727	3.191993	2.668353
3	1	0	-0.686991	2.690952	3.488744
4	1	0	-2.040338	3.729548	3.121591
5	6	0	-0.286684	4.180891	1.967688
6	1	0	0.095626	4.915003	2.681959
7	1	0	-0.830250	4.711076	1.181738
8	1	0	0.566978	3.672506	1.514427
9	6	0	-3.957962	3.331989	1.249894
10	1	0	-3.678210	3.491246	0.207708
11	1	0	-3.708979	4.234523	1.813200
12	1	0	-5.040873	3.197175	1.304500
13	6	0	-3.261014	2.102776	1.814188
14	1	0	-3.583534	1.220889	1.263736
15	1	0	-3.563735	1.936606	2.856149
16	6	0	-1.094132	1.011443	1.540176
17	9	0	0.172021	1.123877	2.030223
18	9	0	-1.649354	-0.057411	2.216782
19	6	0	-0.874034	0.584330	0.067338
20	1	0	-0.246104	1.350630	-0.392952
21	6	0	-2.093038	0.391583	-0.840975
22	9	0	-1.692581	-0.116935	-2.011148
23	9	0	-2.999637	-0.447047	-0.329067
24	9	0	-2.698672	1.555677	-1.095719
25	9	0	-0.193608	-0.610241	0.062852

1Cc_$CHCl_3$_ωB97X-D/6-311++G(d,p) $E = -927.2022971$

Standard orientation:

Center Number	Atomic Number	Atomic Type	Coordinates (Angstroms) X	Y	Z
1	6	0	2.391032	-0.639352	0.458021
2	1	0	2.348525	-1.730108	0.460845
3	1	0	3.263727	-0.371944	-0.143153
4	6	0	1.490353	0.810696	-1.337798
5	1	0	2.124400	0.296142	-2.071884
6	1	0	0.561919	1.063781	-1.849406
7	6	0	0.143883	-0.981123	-0.379874
8	9	0	0.023226	-1.763634	0.722854
9	9	0	0.346700	-1.895984	-1.424001
10	7	0	1.196384	-0.088989	-0.214345
11	6	0	-1.238921	-0.356369	-0.668905
12	1	0	-1.257600	0.094594	-1.661433
13	6	0	-1.686532	0.715186	0.336921
14	9	0	-1.014125	1.855888	0.142448
15	9	0	-1.525300	0.349707	1.608083
16	9	0	-2.985712	0.979752	0.152534
17	9	0	-2.167479	-1.365284	-0.641157
18	6	0	2.155142	2.098427	-0.874790
19	1	0	2.331990	2.748526	-1.734587
20	1	0	1.518444	2.625199	-0.162015
21	1	0	3.120510	1.907783	-0.400505
22	6	0	2.541534	-0.112836	1.877610
23	1	0	2.614207	0.977121	1.881283
24	1	0	1.681482	-0.400370	2.485555
25	1	0	3.443989	-0.522546	2.339257

1Aa_C_5H_5N_ωB97X-D/6-311++G(d,p) $E = -927.2008071$

Standard orientation:

Center Number	Atomic Number	Atomic Type	Coordinates (Angstroms) X	Y	Z
1	6	0	-1.401207	-1.287122	0.531842
2	1	0	-0.506354	-1.895957	0.393632
3	1	0	-1.521136	-1.164132	1.611691
4	6	0	-1.543877	1.194771	0.806851
5	1	0	-0.971289	1.153921	1.743060
6	1	0	-1.245892	2.104358	0.286315
7	6	0	-0.203508	0.228870	-0.997572
8	9	0	-0.563171	1.245056	-1.854989
9	9	0	-0.092976	-0.897519	-1.756695
10	7	0	-1.204694	0.050184	-0.043331
11	6	0	1.254049	0.604954	-0.590548
12	1	0	1.872616	0.615263	-1.490247
13	6	0	1.922946	-0.317942	0.430783
14	9	0	1.970584	-1.578964	-0.010262
15	9	0	1.291507	-0.313127	1.609758
16	9	0	3.179739	0.085648	0.649494
17	9	0	1.251567	1.864274	-0.037799
18	6	0	-3.034187	1.257812	1.110499
19	1	0	-3.248766	2.144160	1.712270
20	1	0	-3.612417	1.314176	0.185360
21	1	0	-3.369129	0.384111	1.674857
22	6	0	-2.602702	-2.014476	-0.058777
23	1	0	-3.520064	-1.440495	0.087427
24	1	0	-2.464095	-2.172735	-1.130152
25	1	0	-2.725074	-2.989495	0.421303

1Ab_C_5H_5N_ωB97X-D/6-311++G(d,p) $E = -927.2034849$

Standard orientation:

Center Number	Atomic Number	Atomic Type	Coordinates (Angstroms) X	Y	Z
1	6	0	1.192164	1.017137	1.255393
2	1	0	0.189107	1.152336	1.653791
3	1	0	1.817895	0.663168	2.085249
4	6	0	2.486314	-0.411565	-0.303224
5	1	0	3.237341	0.031664	0.355068
6	1	0	2.603846	-1.492984	-0.208605
7	6	0	0.234768	-1.025791	0.331206
8	9	0	0.485836	-1.865302	1.426885
9	9	0	0.318419	-1.861704	-0.745577
10	7	0	1.157001	-0.000149	0.193863
11	6	0	-1.266623	-0.672196	0.452680
12	1	0	-1.812663	-1.600457	0.273864
13	6	0	-1.778445	0.364253	-0.555823
14	9	0	-1.417355	0.047232	-1.800312
15	9	0	-1.344253	1.598870	-0.294693
16	9	0	-3.118108	0.399357	-0.513652
17	9	0	-1.586706	-0.220034	1.709139
18	6	0	2.720476	0.021863	-1.742714
19	1	0	3.726342	-0.261754	-2.064059
20	1	0	1.997797	-0.452312	-2.409786
21	1	0	2.617595	1.104859	-1.842022
22	6	0	1.706927	2.351821	0.737750
23	1	0	2.734116	2.277731	0.373087
24	1	0	1.077014	2.715924	-0.075986
25	1	0	1.693909	3.086974	1.545522

1Ac_C_5H_5N_ωB97X-D/6-311++G(d,p) $E = -927.1970861$

Standard orientation:

Center Number	Atomic Number	Atomic Type	Coordinates (Angstroms) X	Y	Z
1	6	0	2.592441	-0.492956	-0.117858
2	1	0	3.426018	-0.013453	0.400115
3	1	0	2.667902	-1.557191	0.102488
4	6	0	1.030090	1.418254	0.277958
5	1	0	0.796437	1.624507	-0.772566
6	1	0	0.149047	1.666670	0.864620
7	6	0	0.363925	-0.942593	0.671725
8	9	0	0.837207	-1.942012	1.508673
9	9	0	0.021133	-1.674996	-0.477329
10	7	0	1.342261	0.001157	0.478637
11	6	0	-0.989955	-0.528603	1.279503
12	1	0	-1.490870	-1.467342	1.518234
13	6	0	-1.976636	0.273211	0.378603
14	9	0	-3.149345	-0.372903	0.320367
15	9	0	-1.551193	0.429913	-0.876839
16	9	0	-2.214695	1.486214	0.881404
17	9	0	-0.788669	0.157147	2.448390
18	6	0	2.161638	2.322742	0.746556
19	1	0	1.838738	3.363160	0.670884
20	1	0	2.417831	2.113598	1.787750
21	1	0	3.060729	2.212834	0.136820
22	6	0	2.701654	-0.263115	-1.622375
23	1	0	1.865892	-0.726498	-2.150167
24	1	0	2.720403	0.801013	-1.870696
25	1	0	3.630631	-0.704684	-1.990550

1Ba_C_5H_5N_ωB97X-D/6-311++G(d,p) $E = -927.2009036$

Standard orientation:

Center Number	Atomic Number	Atomic Type	Coordinates (Angstroms) X	Y	Z
1	6	0	2.229412	-0.674432	-0.877258
2	1	0	1.731960	-1.567686	-1.248737
3	1	0	2.139615	0.091501	-1.656219
4	6	0	2.036536	0.981942	0.982074
5	1	0	1.371426	1.218442	1.813466
6	1	0	3.006588	0.761538	1.434446
7	6	0	0.182941	-0.526608	0.496618
8	9	0	-0.138913	-0.554714	1.832796
9	9	0	-0.091476	-1.766244	0.009791
10	7	0	1.549248	-0.253731	0.353767
11	6	0	-0.802014	0.485387	-0.142226
12	1	0	-0.687847	1.444932	0.366912
13	6	0	-2.292914	0.126666	-0.114567
14	9	0	-2.727802	-0.049256	1.135102
15	9	0	-2.572370	-0.974655	-0.813872
16	9	0	-3.000965	1.131155	-0.653613
17	9	0	-0.456265	0.638561	-1.466132
18	6	0	2.172954	2.190710	0.057003
19	1	0	2.485350	3.056097	0.645767
20	1	0	2.927537	2.026103	-0.715320
21	1	0	1.233763	2.442344	-0.441189
22	6	0	3.696742	-0.988624	-0.620783
23	1	0	4.260116	-0.101435	-0.321128
24	1	0	3.798052	-1.743560	0.162379
25	1	0	4.151160	-1.373808	-1.536326

1Bb_C_5H_5N_ωB97X-D/6-311++G(d,p) $E = -927.2024324$

Standard orientation:

Center Number	Atomic Number	Atomic Type	Coordinates (Angstroms) X	Y	Z
1	6	0	1.887530	1.298183	0.748379
2	1	0	1.040302	1.982822	0.750046
3	1	0	2.186724	1.155002	1.795247
4	6	0	2.492564	-1.050642	0.213828
5	1	0	3.284053	-0.722829	0.892839
6	1	0	2.063910	-1.949012	0.662299
7	6	0	0.174351	-0.404695	0.380582
8	9	0	-0.075540	-0.819836	1.702354
9	9	0	-0.068243	-1.527433	-0.356617
10	7	0	1.473126	0.016349	0.165459
11	6	0	-0.913878	0.654623	0.096655
12	1	0	-0.967110	1.350159	0.935232
13	6	0	-2.327784	0.084901	-0.104479
14	9	0	-2.690865	-0.713365	0.900017
15	9	0	-2.447100	-0.593177	-1.246203
16	9	0	-3.201750	1.101960	-0.153554
17	9	0	-0.609012	1.349644	-1.046732
18	6	0	3.065397	-1.373669	-1.158570
19	1	0	3.830296	-2.150191	-1.072896
20	1	0	2.280464	-1.734483	-1.826151
21	1	0	3.520168	-0.489857	-1.610927
22	6	0	3.017641	1.944087	-0.039304
23	1	0	3.924936	1.336120	-0.023189
24	1	0	2.721807	2.098077	-1.079374
25	1	0	3.260228	2.912887	0.402734

1Bc_C_5H_5N_ωB97X-D/6-311++G(d,p) $E = -927.2025127$

Standard orientation:

Center Number	Atomic Number	Atomic Type	Coordinates (Angstroms) X	Y	Z
1	6	0	-2.641554	-0.425119	0.681603
2	1	0	-2.277538	-1.302647	1.215344
3	1	0	-3.139156	0.205030	1.424879
4	6	0	-1.689244	1.744570	-0.051981
5	1	0	-0.953828	2.128778	-0.761544
6	1	0	-2.658473	1.861148	-0.541553
7	6	0	-0.243534	-0.194649	0.439667
8	9	0	-0.194973	-1.536135	0.154875
9	9	0	0.045545	-0.149522	1.807545
10	7	0	-1.493152	0.300859	0.114088
11	6	0	0.932359	0.487866	-0.296133
12	1	0	0.637047	0.712402	-1.322643
13	6	0	2.213842	-0.359740	-0.377585
14	9	0	2.067497	-1.360271	-1.250491
15	9	0	2.585788	-0.876494	0.794589
16	9	0	3.217472	0.411976	-0.813824
17	9	0	1.261787	1.657407	0.343216
18	6	0	-1.655666	2.567953	1.234390
19	1	0	-1.858873	3.615734	1.000313
20	1	0	-2.413318	2.232042	1.946044
21	1	0	-0.679934	2.508247	1.717768
22	6	0	-3.624837	-0.850809	-0.399478
23	1	0	-3.998906	0.010489	-0.958822
24	1	0	-3.147609	-1.536954	-1.102429
25	1	0	-4.483134	-1.354619	0.052077

1Ca_C_5H_5N_ωB97X-D/6-311++G(d,p) $E = -927.1984853$

Standard orientation:

Center Number	Atomic Number	Atomic Type	Coordinates (Angstroms) X	Y	Z
1	6	0	-1.671164	-1.374340	-0.841449
2	1	0	-2.714142	-1.248381	-1.148849
3	1	0	-1.075029	-1.346490	-1.752310
4	6	0	-2.428555	0.288823	0.812003
5	1	0	-3.076213	0.914932	0.182421
6	1	0	-3.024384	-0.583543	1.085706
7	6	0	-0.283974	0.619691	-0.377319
8	9	0	0.630520	-0.056843	-1.120003
9	9	0	0.412144	1.083803	0.719863
10	7	0	-1.302606	-0.248530	0.033310
11	6	0	-0.674090	1.910271	-1.140670
12	1	0	-1.330704	2.508346	-0.504926
13	6	0	-1.394613	1.700332	-2.477144
14	9	0	-2.597151	1.143016	-2.286884
15	9	0	-0.711543	0.925520	-3.322721
16	9	0	-1.585372	2.880764	-3.075236
17	9	0	0.459940	2.640526	-1.406976
18	6	0	-2.061735	1.030841	2.089919
19	1	0	-2.981398	1.244390	2.639454
20	1	0	-1.411671	0.421733	2.720568
21	1	0	-1.561204	1.981458	1.900223
22	6	0	-1.479954	-2.713576	-0.143819
23	1	0	-0.425732	-2.874275	0.091912
24	1	0	-2.050613	-2.757227	0.787672
25	1	0	-1.821314	-3.526802	-0.789737

1Cb_C_5H_5N_ωB97X-D/6-311++G(d,p) $E = -927.1977361$

Center Number	Atomic Number	Atomic Type	Coordinates (Angstroms) X	Y	Z
1	7	0	-1.796715	2.212564	1.737631
2	6	0	-1.210213	3.192702	2.672097
3	1	0	-0.691496	2.691365	3.492804
4	1	0	-2.045407	3.728265	3.124469
5	6	0	-0.290395	4.183291	1.973725
6	1	0	0.091032	4.915544	2.690318
7	1	0	-0.834245	4.715626	1.189422
8	1	0	0.563233	3.676358	1.518688
9	6	0	-3.954323	3.335437	1.238925
10	1	0	-3.670100	3.493040	0.197612
11	1	0	-3.705616	4.237750	1.802539
12	1	0	-5.037612	3.202400	1.289472
13	6	0	-3.262522	2.105590	1.807898
14	1	0	-3.583989	1.223555	1.257101
15	1	0	-3.570335	1.942043	2.848581
16	6	0	-1.095147	1.013450	1.542187
17	9	0	0.170113	1.123161	2.035507
18	9	0	-1.651691	-0.058389	2.218945
19	6	0	-0.872149	0.583227	0.070696
20	1	0	-0.241639	1.346303	-0.390803
21	6	0	-2.088896	0.386904	-0.839552
22	9	0	-1.685860	-0.127173	-2.006783
23	9	0	-2.995605	-0.451989	-0.326134
24	9	0	-2.695417	1.548198	-1.099997
25	9	0	-0.192984	-0.613824	0.072098

1Cc_C_5H_5N_ωB97X-D/6-311++G(d,p) $E = -927.2037732$

Standard orientation:

Center Number	Atomic Number	Atomic Type	Coordinates (Angstroms) X	Y	Z
1	6	0	2.394110	-0.618383	0.498283
2	1	0	2.341354	-1.705907	0.575605
3	1	0	3.262776	-0.400569	-0.127720
4	6	0	1.492497	0.717889	-1.383332
5	1	0	2.118200	0.148192	-2.082519
6	1	0	0.563697	0.943722	-1.906429
7	6	0	0.138421	-0.993740	-0.298410
8	9	0	0.011515	-1.693512	0.859738
9	9	0	0.331809	-1.988702	-1.271310
10	7	0	1.197758	-0.102215	-0.199168
11	6	0	-1.239998	-0.382828	-0.633723
12	1	0	-1.258307	-0.010169	-1.658012
13	6	0	-1.677562	0.764539	0.289348
14	9	0	-0.996791	1.881952	0.014073
15	9	0	-1.518216	0.490028	1.584834
16	9	0	-2.975383	1.024497	0.088413
17	9	0	-2.175801	-1.381144	-0.525091
18	6	0	2.172138	2.026957	-1.011521
19	1	0	2.353143	2.612534	-1.915546
20	1	0	1.542841	2.609413	-0.336289
21	1	0	3.136507	1.858428	-0.527150
22	6	0	2.565146	0.001161	1.877382
23	1	0	2.646642	1.088167	1.805556
24	1	0	1.711194	-0.238232	2.514260
25	1	0	3.470624	-0.383986	2.353777

Table S1. Calculated [ωB97X-D/6-311++g(d,p)] spin-spin coupling constants and F–C–C–F dihedral angles, in implicit cyclohexane solvent.

Conformer	$^{1}J_{C,Fpro\text{-}S}$	$^{1}J_{C,Fpro\text{-}R}$	$^{3}J_{F\text{-}2,Fpro\text{-}S}$	$^{3}J_{F\text{-}2,Fpro\text{-}R}$	F-C-C-F(pro-*S*)	F-C-C-F(pro-*R*)
**1Aa**	-260.7	-253.1	-20.2	-14.09	57.0	169.2
**1Ab**	-283.3	-235.3	-21.5	-14.69	49.4	159.5
**1Ac**	-229.9	-251.2	-17.4	-23.22	70.4	177.5
**1Ba**	-250.0	-262.8	-27.1	-14.89	173.4	-72.2
**1Bb**	-265.2	-247.1	-30.2	-11.43	163.0	-85.5
**1Bc**	-236.3	-280.2	-16.7	-27.84	153.6	-45.0
**1Ca**	-259.4	-280.9	-9.7	-17.18	-46.8	64.5
**1Cb**	-298.7	-260.1	-25.3	-17.8	-51.2	60.2
**1Cc**	-249.8	-279.7	-8.2	-16.75	-48.0	63.2

Table S2. Calculated [ωB97X-D/6-311++g(d,p)] spin-spin coupling constants and F–C–C–F dihedral angles, in implicit pyridine solvent.

Conformer	$^{1}J_{\mathrm{C,Fpro}\text{-}S}$	$^{1}J_{\mathrm{C,Fpro}\text{-}R}$	$^{3}J_{\mathrm{F\text{-}2,Fpro}\text{-}S}$	$^{3}J_{\mathrm{F\text{-}2,Fpro}\text{-}R}$	F-C-C-F(pro-*S*)	F-C-C-F(pro-*R*)
**1Aa**	-258.0	-252.7	-20.4	-13.1	57.1	168.8
**1Ab**	-281.2	-234.4	-22.1	-14.3	50.7	159.7
**1Ac**	-225.8	-252.8	-16.8	-24.1	71.3	177.7
**1Ba**	-248.7	-260.3	-25.7	-15.4	175.2	-71.2
**1Bb**	-264.8	-243.1	-30.1	-12.1	166.8	-82.8
**1Bc**	-231.9	-275.8	-18.0	-30.3	155.8	-45.4
**1Ca**	-258.4	-255.1	-22.3	-31.7	-56.0	55.5
**1Cb**	-296.6	-259.9	-27.3	-18.2	-50.8	60.1
**1Cc**	-245.7	-278.5	-8.3	-17.1	-48.7	61.3

Table S3. Calculated $^3J_{H2,F1}$ coupling constants (in Hz) and H–C–C–F(1) dihedral angles (in degrees) for the optimized geometries of **1**, in cyclohexane and pyridine implicit solvents.

Conf.	Cyclohexane				Pyridine			
	$^3J_{H,Fpro\text{-}R}$	$^3J_{H,Fpro\text{-}S}$	H-C-C-$F_{pro\text{-}R}$	H-C-C-$F_{pro\text{-}S}$	$^3J_{H,Fpro\text{-}R}$	$^3J_{H,Fpro\text{-}S}$	H-C-C-$F_{pro\text{-}R}$	H-C-C-$F_{pro\text{-}S}$
**1Aa**	-0.1	-1.5	50.9	-61.3	-0.2	-1.5	50.4	-61.2
**1Ab**	9.9	-0.3	41.3	-68.9	9.9	-0.3	41.8	-67.2
**1Ac**	2.8	9.9	59.5	-48.7	1.8	10.8	61.5	-44.9
**1Ba**	15.6	0.2	169.6	55.2	15.9	-0.3	171.0	57.3
**1Bb**	13.9	1.0	154.3	42.9	14.3	0.5	157.3	46.9
**1Bc**	8.3	-0.9	164.6	83.8	8.6	-0.7	-165.1	84.5
**1Ca**	0.8	9.8	-53.8	-165.0	0.5	14.0	-62.8	-174.3
**1Cb**	6.8	8.9	-56.9	-168.3	6.8	9.1	-57.0	-167.9
**1Cc**	0.1	9.3	-55.0	-166.2	-0.2	9.5	-56.8	-166.8

Table S4. Relative nuclear-electronic energies (in kcal mol^{-1} and % population in parenthesis) and molecular dipole moments (in D) for the conformers of **1**, calculated at the MP2/6-311++g(d,p) level.

Conf.	Cyclohexane		Chloroform		Pyridine	
	G^0rel (%)	μ	G^0rel (%)	μ	G^0rel (%)	μ
**1Aa**	2.3 (1)	1.5	2.3 (1)	1.6	2.4 (1)	1.7
**1Ab**	0.0 (65)	2.1	0.0 (49)	2.2	0.2 (37)	2.2
**1Ac**	5.9 (0)	2.4	5.8 (0)	2.5	5.9 (0)	2.6
**1Ba**	3.1 (0)	4.5	2.8 (0)	4.8	2.8 (0)	5.0
**1Bb**	1.6 (5)	4.5	1.2 (6)	4.8	1.1 (8)	5.0
**1Bc**	1.9 (3)	4.7	1.5 (4)	5.1	1.4 (4)	5.4
**1Ca**	4.7 (0)	4.4	4.3 (0)	4.8	4.2 (0)	5.1
**1Cb**	4.1 (0)	4.2	3.9 (0)	4.5	4.0 (0)	4.6
**1Cc**	0.6 (26)	4.5	0.1 (40)	4.9	0.0 (50)	5.2

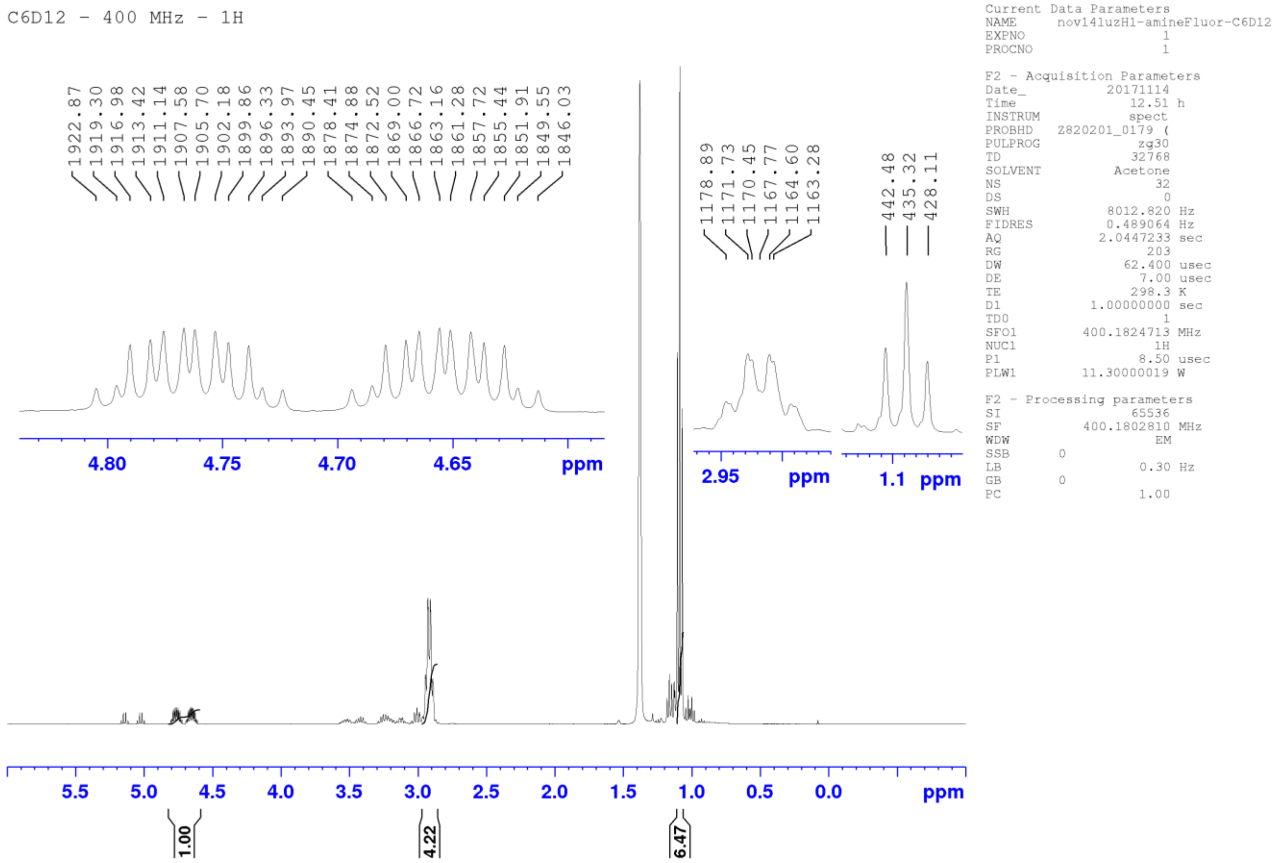

Figure S1. ^{1}H NMR spectrum of **1** in 10 mg mL^{-1} cyclohexane-d_{12} solution.

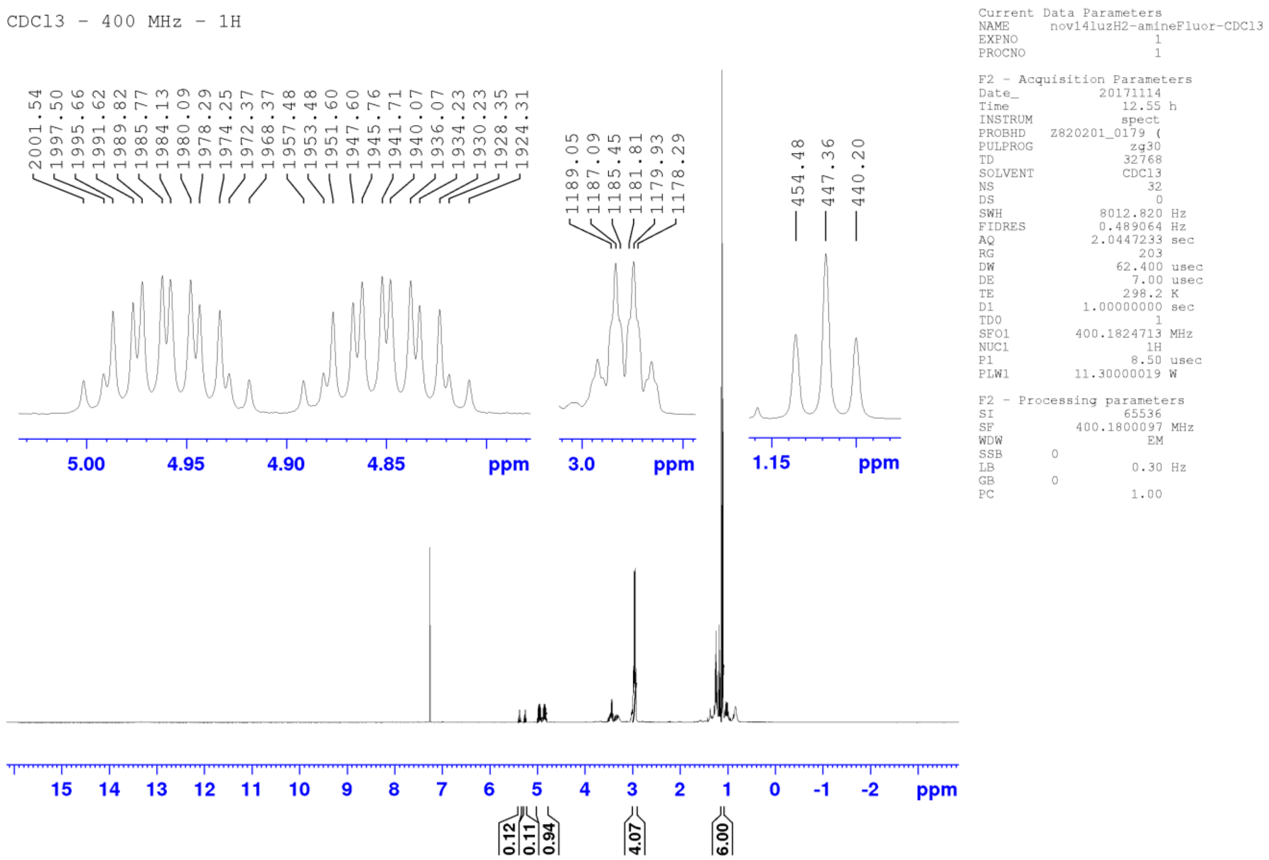

Figure S2. ^{1}H NMR spectrum of **1** in 10 mg mL^{-1} $CDCl_3$ solution.

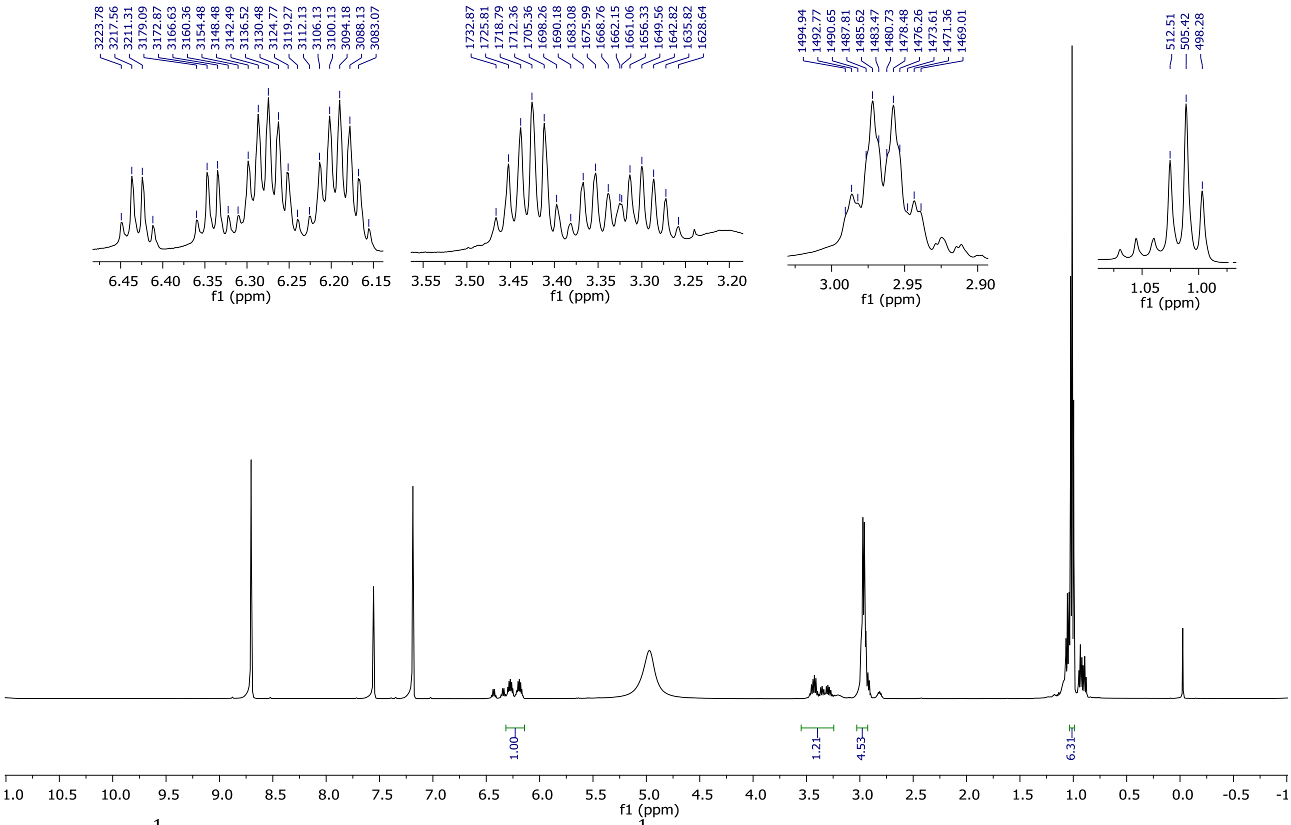

Figure S3. ^{1}H NMR spectrum of **1** in 10 mg mL^{-1} pyridine-d_5 solution.

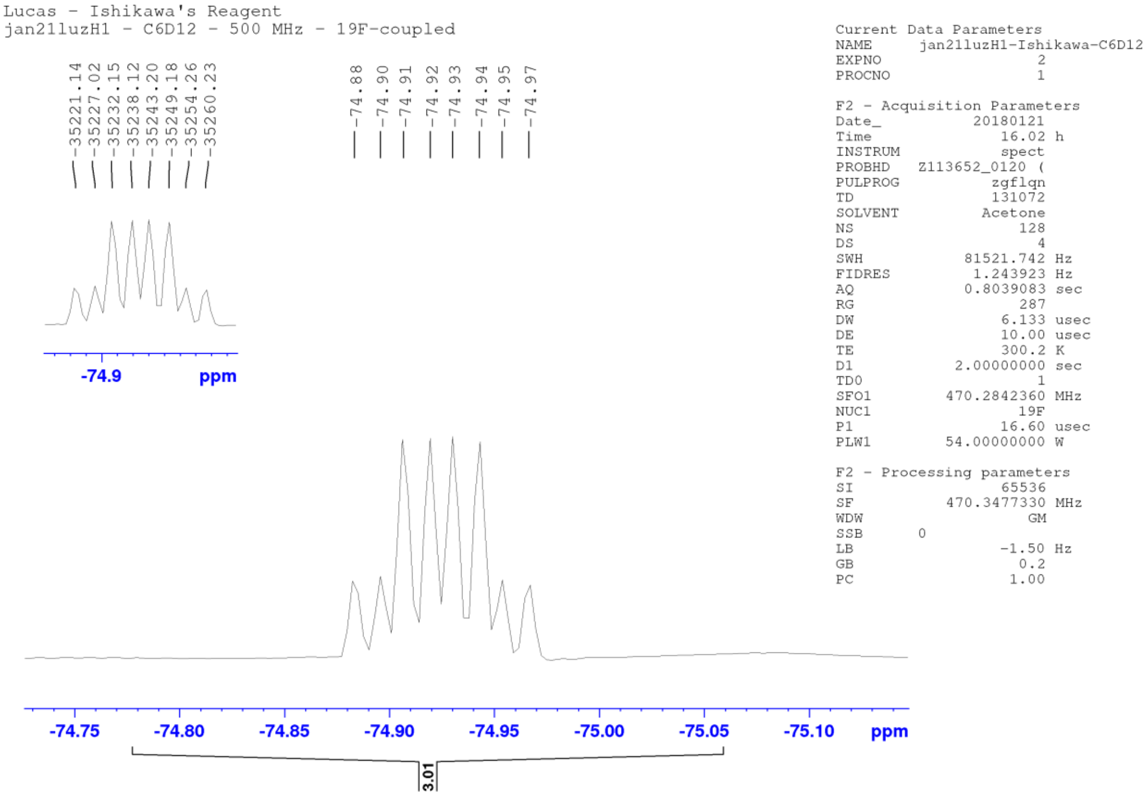

Figure S4. ^{19}F NMR spectrum (expansion in the CF_3 region) of **1** in 10 mg mL^{-1} cyclohexane-d_{12} solution.

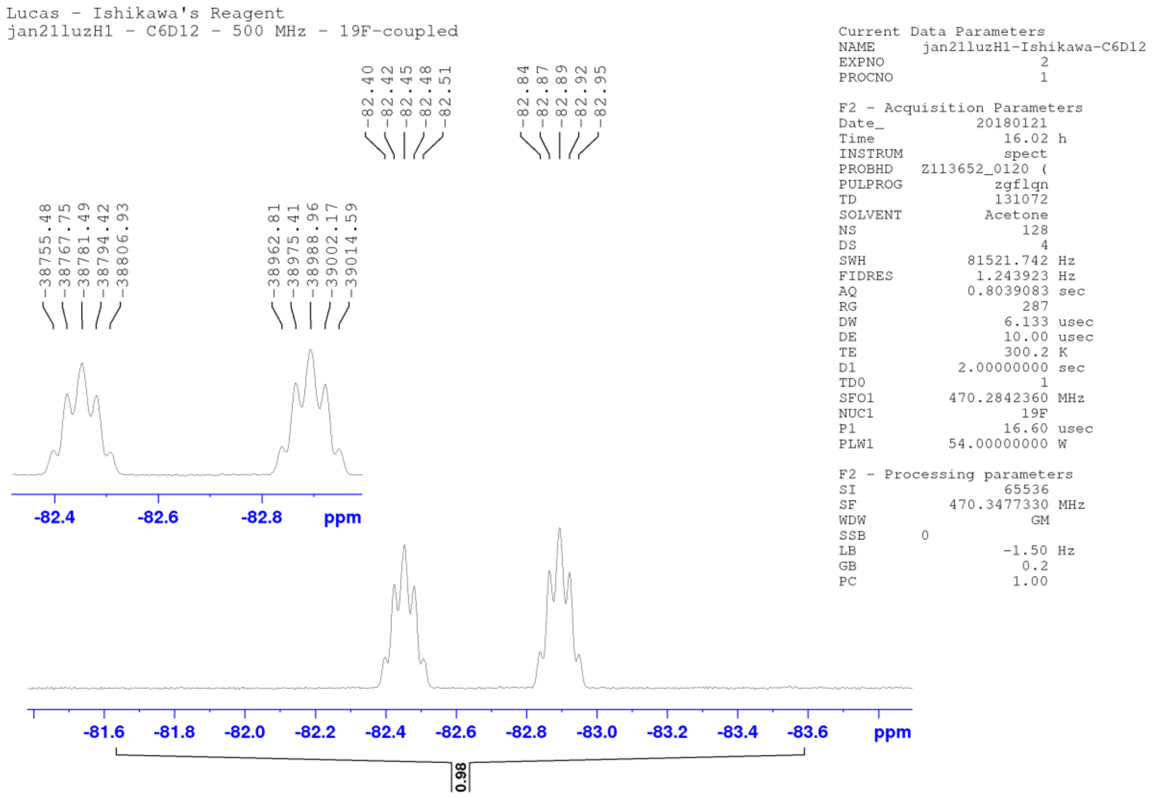

Figure S5. ^{19}F NMR spectrum (expansion in the $F_{pro\text{-}S}$ region) of **1** in 10 mg mL^{-1} cyclohexane-d_{12} solution.

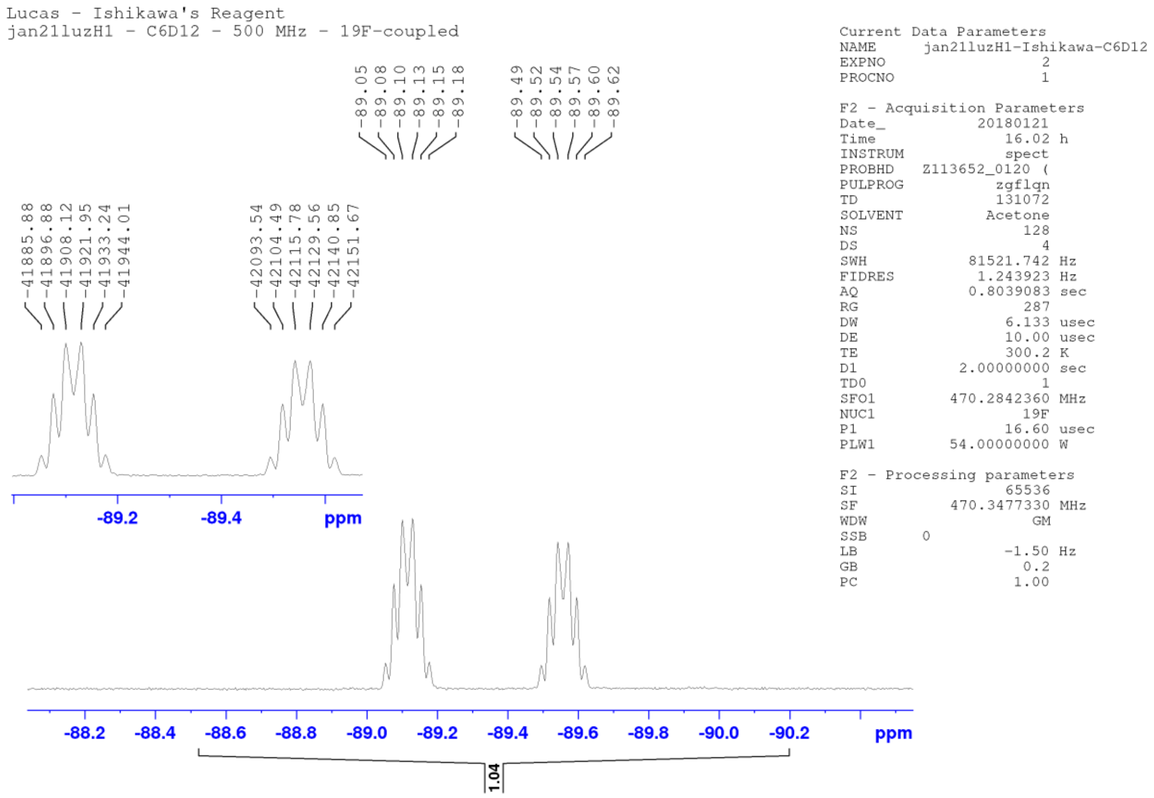

Figure S6. ^{19}F NMR spectrum (expansion in the $F_{pro\text{-}R}$ region) of **1** in 10 mg mL^{-1} cyclohexane-d_{12} solution.

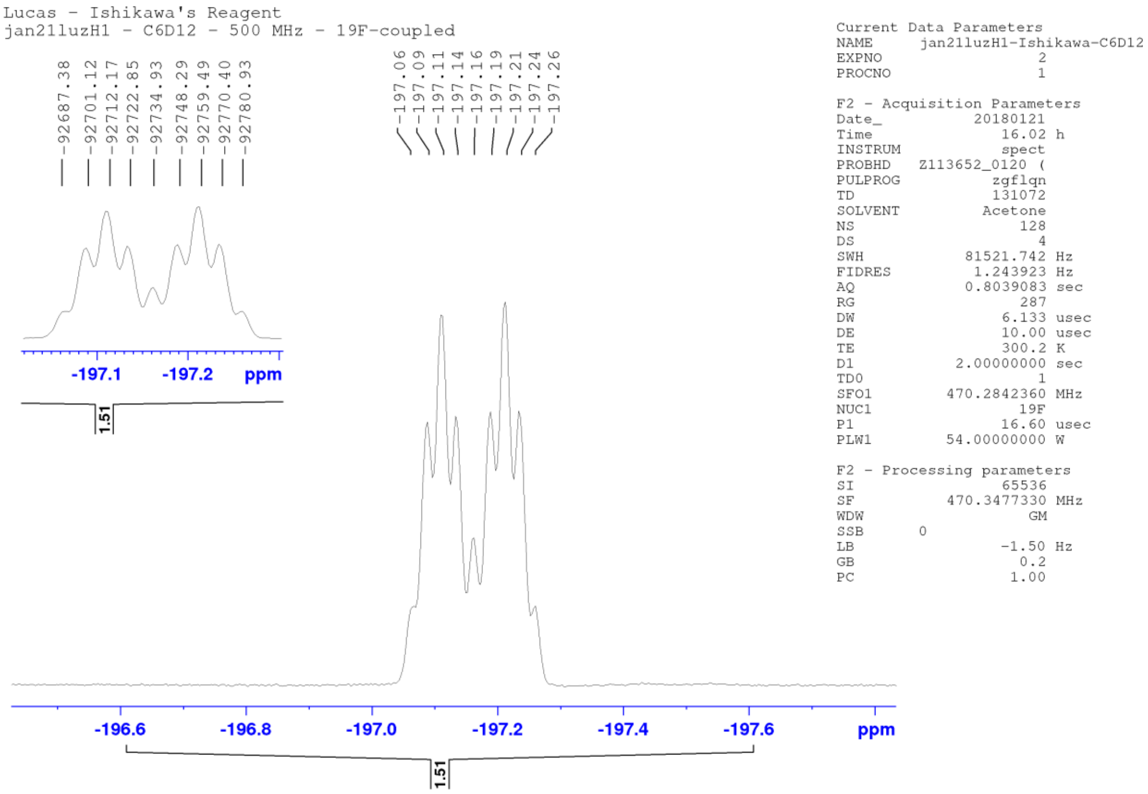

Figure S7. ^{19}F NMR spectrum (expansion in the F-2 region) of **1** in 10 mg mL^{-1} cyclohexane-d_{12} solution.

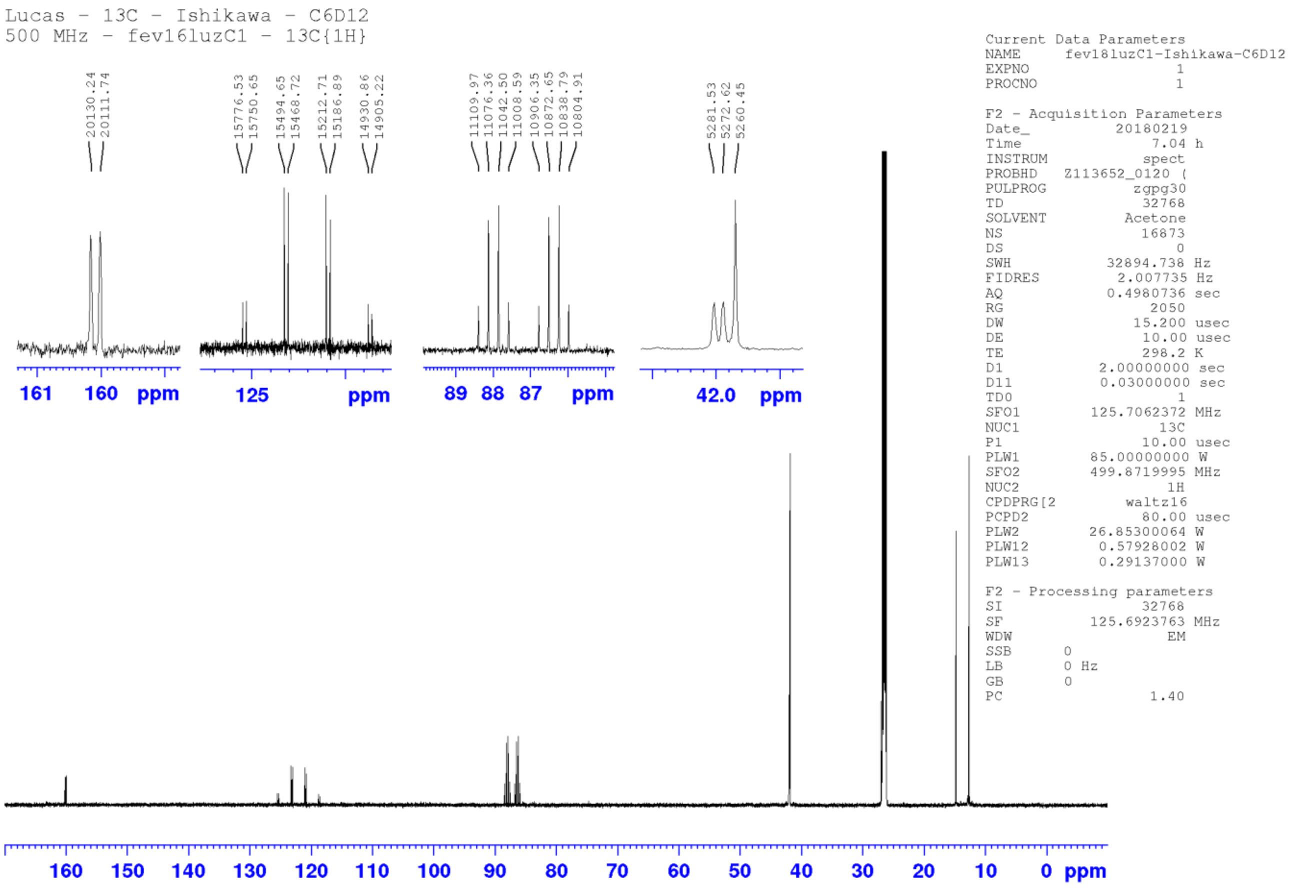

Figure S8. ^{13}C NMR spectrum of **1** in 10 mg mL^{-1} cyclohexane-d_{12} solution.

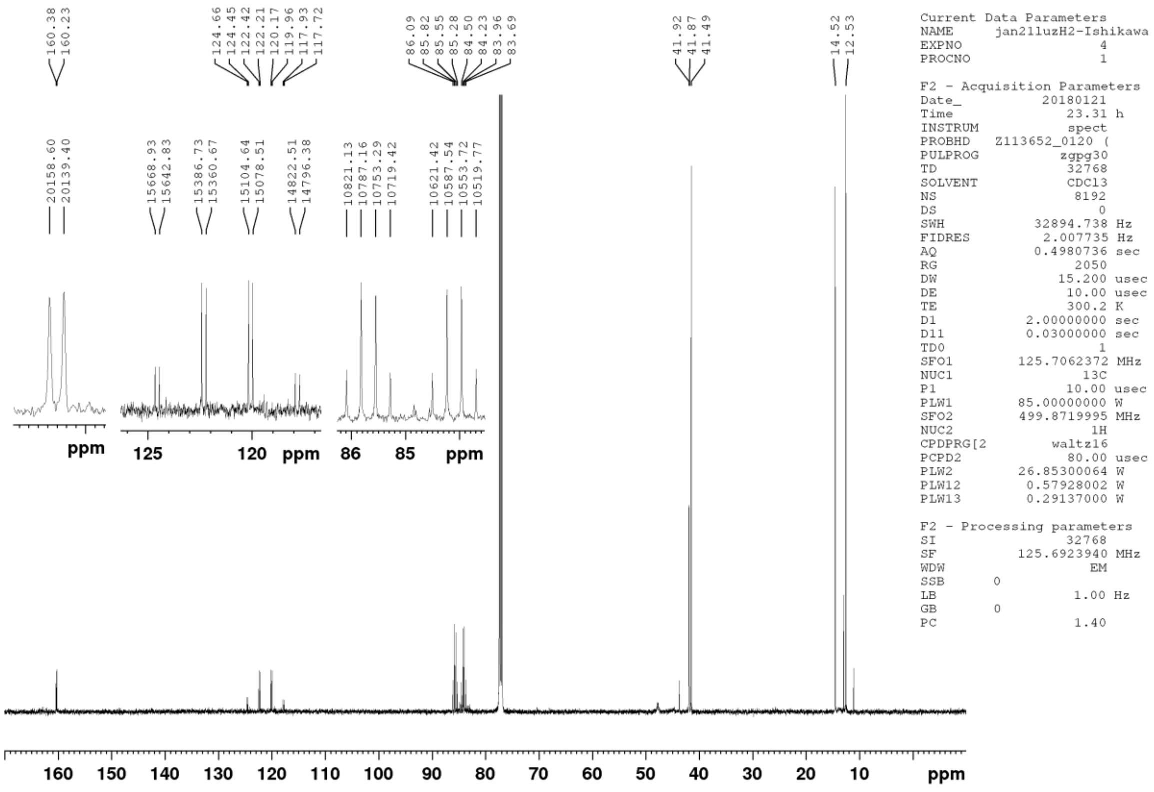

Figure S9. ^{13}C NMR spectrum of **1** in 10 mg mL^{-1} $CDCl_3$ solution.

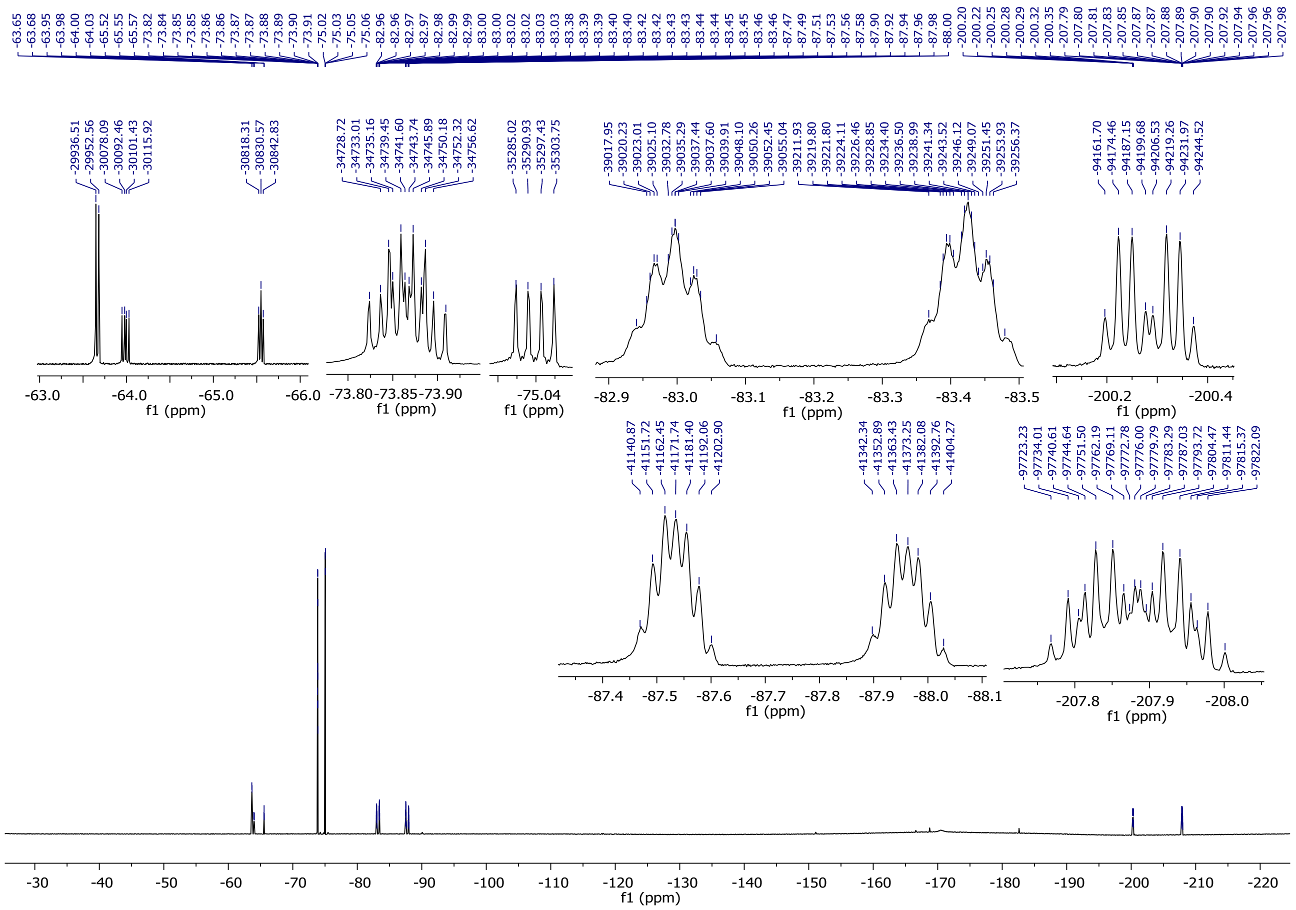

Figure S10. ^{19}F NMR spectrum of **1** in 10 mg mL^{-1} pyridine-d_5 solution.

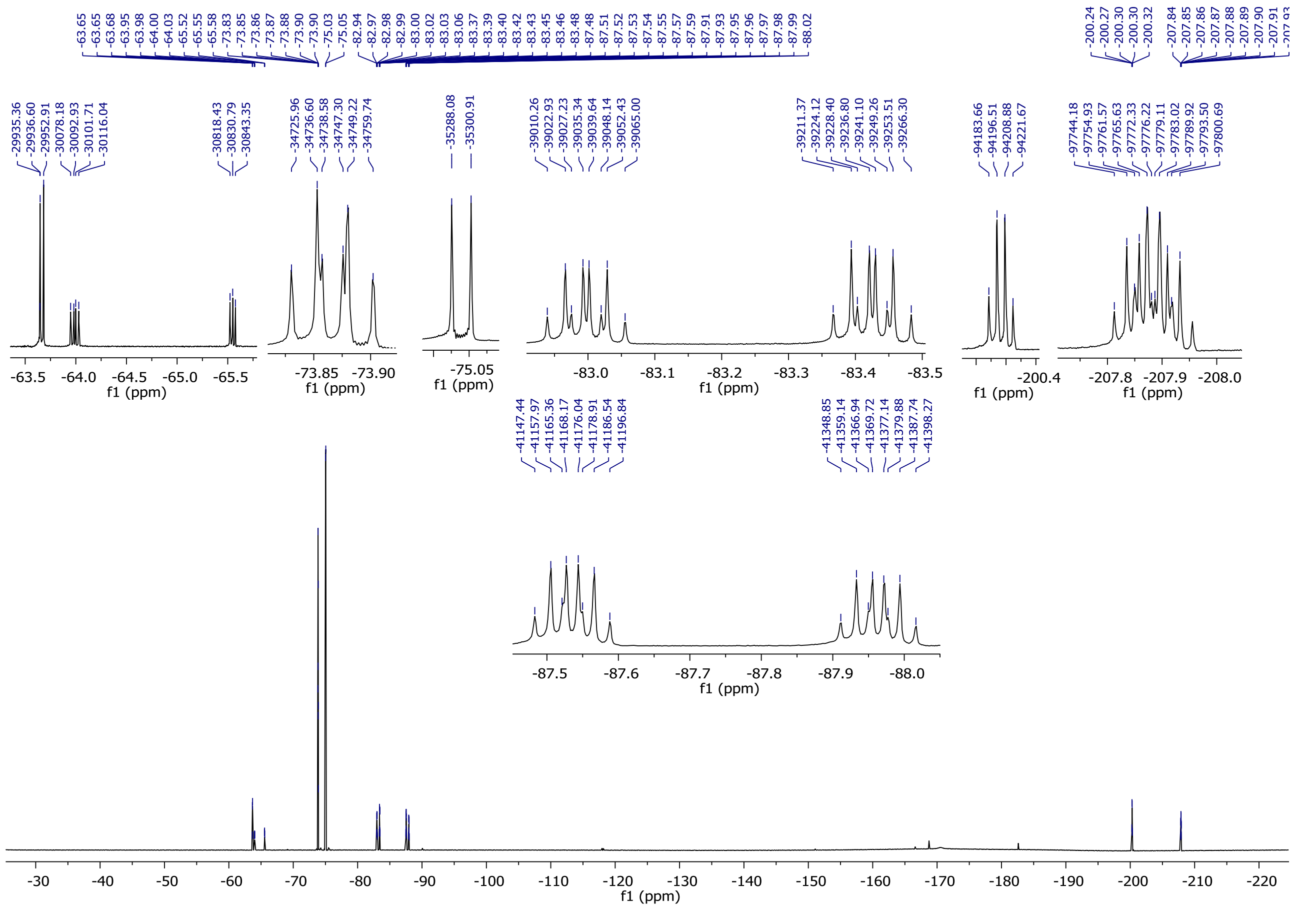

Figure S11. ${}^{19}F\{{}^{1}H\}$ NMR spectrum of **1** in 10 mg mL^{-1} pyridine-d_5 solution.

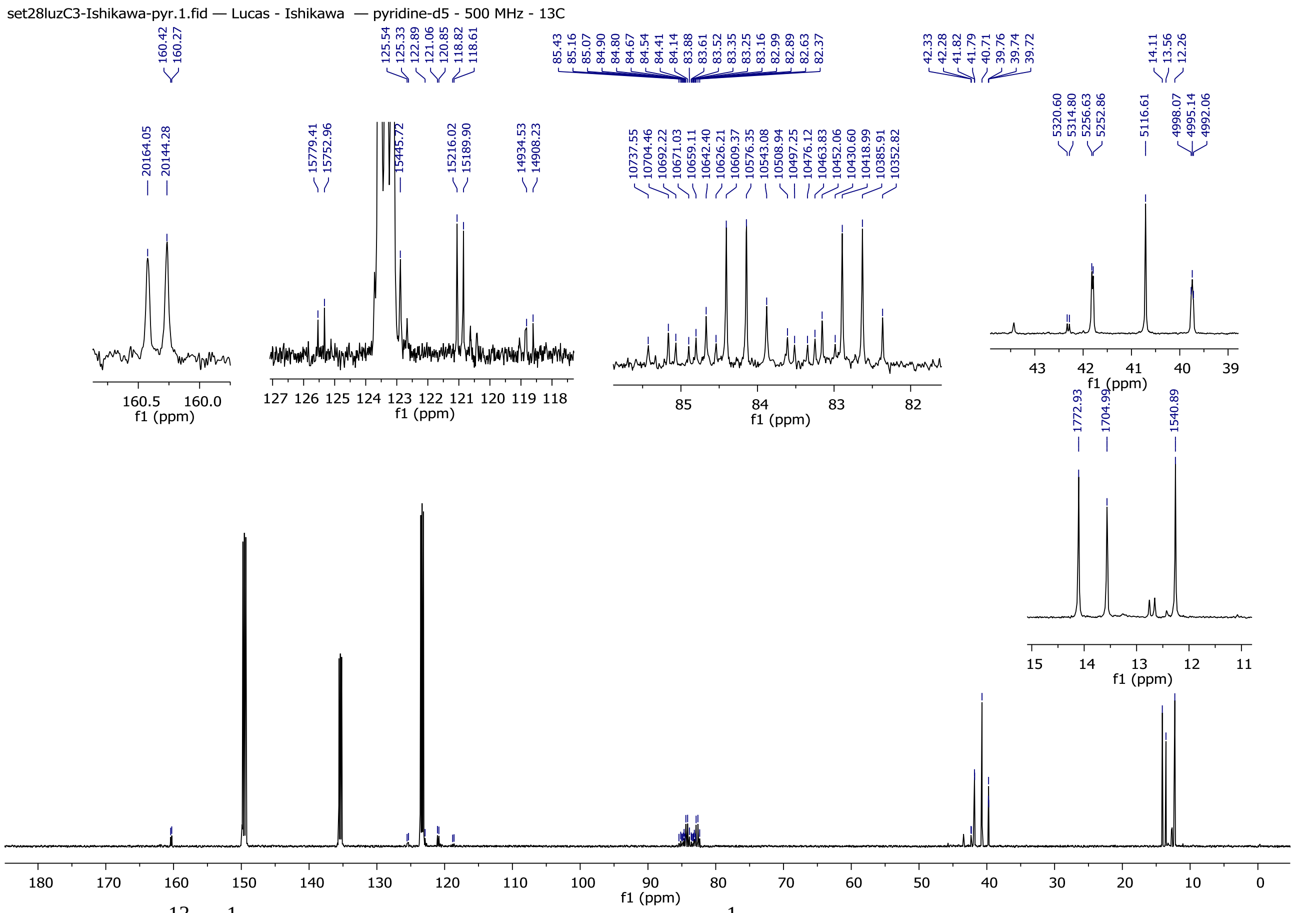

Figure S12. $^{13}C\{^{1}H\}$ NMR spectrum of **1** in 10 mg mL^{-1} pyridine-d_5 solution.
